# Identification of predictive biomarkers for endometrial cancer diagnosis and treatment response monitoring using plasma metabolome profiling

**DOI:** 10.1186/s40170-023-00317-z

**Published:** 2023-10-11

**Authors:** Eiji Hishinuma, Muneaki Shimada, Naomi Matsukawa, Yoshiko Shima, Bin Li, Ikuko N. Motoike, Yusuke Shibuya, Tatsuya Hagihara, Shogo Shigeta, Hideki Tokunaga, Daisuke Saigusa, Kengo Kinoshita, Seizo Koshiba, Nobuo Yaegashi

**Affiliations:** 1 Advanced Research Center for Innovations in Next-Generation Medicine, Tohoku University, Sendai, 980-8573 Japan; 2 Tohoku Medical Megabank Organization, Tohoku University, Sendai, 980-8573 Japan; 3 Department of Gynecology and Obstetrics, Graduate School of Medicine, Tohoku University, Sendai, 980-8574 Japan; 4 Systems Bioinformatics, Graduate School of Information Sciences, Tohoku University, Sendai, 980-8579 Japan; 5 Laboratory of Biomedical and Analytical Sciences, Faculty of Pharma-Science, Teikyo University, Tokyo, 173-8605 Japan

**Keywords:** Endometrial cancer, Metabolome analysis, Biomarker, Mass spectrometry

## Abstract

**Background:**

Endometrial cancer (EMC) is the most common female genital tract malignancy with an increasing prevalence in many countries including Japan, a fact that renders early detection and treatment necessary to protect health and fertility. Although early detection and treatment are necessary to further improve the prognosis of women with endometrial cancer, biomarkers that accurately reflect the pathophysiology of EMC patients are still unclear. Therefore, it is clinically critical to identify biomarkers to assess diagnosis and treatment efficacy to facilitate appropriate treatment and development of new therapies for EMC.

**Methods:**

In this study, wide-targeted plasma metabolome analysis was performed to identify biomarkers for EMC diagnosis and the prediction of treatment responses. The absolute quantification of 628 metabolites in plasma samples from 142 patients with EMC was performed using ultra-high-performance liquid chromatography with tandem mass spectrometry.

**Results:**

The concentrations of 111 metabolites increased significantly, while the concentrations of 148 metabolites decreased significantly in patients with EMC compared to healthy controls. Specifically, LysoPC and TGs, including unsaturated fatty acids, were reduced in patients with stage IA EMC compared to healthy controls, indicating that these metabolic profiles could be used as early diagnostic markers of EMC. In contrast, blood levels of amino acids such as histidine and tryptophan decreased as the risk of recurrence increased and the stages of EMC advanced. Furthermore, a marked increase in total TG and a decrease in specific TGs and free fatty acids including polyunsaturated fatty acids levels were observed in patients with EMC. These results suggest that the polyunsaturated fatty acids in patients with EMC are crucial for disease progression.

**Conclusions:**

Our data identified specific metabolite profiles that reflect the pathogenesis of EMC and showed that these metabolites correlate with the risk of recurrence and disease stage. Analysis of changes in plasma metabolite profiles could be applied for the early diagnosis and monitoring of the course of treatment of EMC patients.

**Supplementary Information:**

The online version contains supplementary material available at 10.1186/s40170-023-00317-z.

## Background

Endometrial cancer (EMC) is the most common malignancy of the female genital tract in Japan and other developed countries [1]. Its incidence in Japan is increasing annually, reaching more than 17,000 cases in 2019 [2]. EMC is often associated with atypical genital bleeding, so many cases are detected at a relatively early stage. According to the 2019 Patient Annual Report of the Japanese Society of Obstetrics and Gynecology (JSOG) of 12,631 patients with uterine cancer, 7190 (56.9%) were diagnosed to be stage IA [3]. The 5-year overall survival rate for patients with stage IA EMC was 95.3%, based on the 2014 Annual Report on Treatment of JSOG. The basic treatment strategy for patients with stage I–III EMC is surgery, with or without adjuvant therapy, depending on the risk for recurrence evaluated by biopsy of the surgical specimen. The treatment guidelines of the Japanese Society of Gynecologic Oncology recommend no additional postoperative treatment for patients with low-risk recurrence, whereas chemotherapy or radiotherapy is recommended as postoperative treatment for patients with intermediate- or high-risk recurrence [4]. According to the JSOG annual patient report for 2019, 5933 (82.5%) of 7190 patients with stage IA EMC completed their treatment with surgical therapy alone [3].

Approximately 80% of EMCs are grade 1 or 2 endometrioid carcinomas, whose carcinogenesis is associated with estrogen exposure, with obesity being one of the most significant risk factors for this low-grade subtype [5–7]. However, another subtype less associated with estrogen, primarily pathologically diagnosed as grade 3 endometrioid carcinoma, serous, or clear cell carcinoma, shows more aggressive clinical behavior and is more common in the elderly. Recently, according to The Human Cancer Genome Atlas, four classifications of EMC have been proposed based on the results of genetic analysis: polymerase ε (POLE) type (ultramutated), microsatellite instability (MSI) type (hypermutated), copy number low (CN-L) type (endometrioid-like), and copy number high (CN-H) type (serous-like) [8].

Metabolomics is a discipline that involves the comprehensive analysis of metabolites. Unlike genomics which focuses on the genetic information within the cell, metabolomics is influenced by a variety of external factors, including age, sex, environmental factors such as diet and smoking, and changes in gut microbiota [9, 10]. Therefore, it is considered the most accurate reflection of the physiological and pathological changes in an individual’s body. Cancer cells undergo an independent metabolic reprogramming resulting from alterations in various metabolic pathways, including the Warburg effect and glutaminolysis [11]. Exploiting this property, several biomarkers targeting changes in metabolism specific to cancer cells have been developed [12–14]. However, the metabolite biomarkers in previous reports are inconsistent, while no effective biomarkers have yet been identified for the early diagnosis or for monitoring EMC treatment response.

Extensive metabolome analysis has been performed in our previous studies using the MxP^®^ Quant 500 kit to identify novel biomarkers and metabolites as potential targets in the diagnosis of epithelial ovarian and cervical cancer patients and in the prediction of chemotherapy and radiotherapy sensitivity and prognosis [15, 16]. MxP^®^ Quant 500 kit is an ultra-high-performance liquid chromatography-tandem mass spectrometry (UHPLC-MS/MS) wide-targeted metabolome analysis kit that can quantify 628 metabolites with high reproducibility, useful for biomarker discovery. The aim of this study is to profile metabolites in the plasma of patients with EMC using the MxP^®^ Quant 500 kit and to identify new biomarkers that could be potential targets for diagnosing EMC and monitoring disease progression.

## Methods

### Study design and sample collection

From a total of 142 patients with EMC, 108 had stage I, eight had stage II, 12 had stage III, and 14 had stage IV EMC. Histological diagnosis revealed 118 cases with endometrioid carcinoma, nine with serous carcinoma, four with clear cell carcinoma, and 11 with other histological types of carcinoma. In this study, the risk of recurrence classification was based on the pathological final diagnosis after surgery in 142 patients in the study according to the treatment guidelines of the Japan Society of Gynecologic Oncology [4]. As a result, 142 patients were divided into the following recurrent-risk groups; 64 with low-risk, 35 with moderate-risk, 42 with high-risk, and 1 with unknown. In addition, 10 patients were relapsed within the study period. Plasma samples were collected before the first EMC treatment, as part of a clinical biobank project of the Personalized Medicine Center of Tohoku University Hospital and stored in the clinical biobank of the Advanced Research Center for Innovations in Next Generation Medicine (INGEM).

The study was approved by the Ethics Committee of the Tohoku University School of Medicine (approval number 2017–1-346; approved on August 8, 2017) and Tohoku Medical Megabank Organization (ToMMo), Tohoku University (approval number 2018–4-059; approved on October 24, 2018). All patients provided written informed consent before participating in the study. The study was conducted in accordance with the Declaration of Helsinki. Plasma samples from patients with EMC were collected and stored according to the ToMMo cohort protocol [17–20]. The plasma samples were aliquoted into storage tubes and stored at − 80 °C until analysis.

### Materials

Pooled normal human plasma was purchased from Innovative Research (Novi, MI, USA; Lot 26,393) and was used for global quality control (gQC). The other chemicals and reagents used are described in previous studies [15, 16, 21, 22].

### Sample preparation and metabolome analysis

Targeted metabolomic analysis was performed as previously described [15, 16, 21, 22], using an ACQUITY UPLC connected to a triple-quadrupole mass spectrometer (Xevo TQ-XS, Waters Corporation, Milford, MA, USA), and a MxP^®^ Quant 500 kit (Biocrates Life Science AG). Plasma samples of 10 µL were used, and sample preparation and measurements were performed according to the MxP^®^ Quant 500 kit manual. Metabolite concentrations were calculated using exported raw data files with the MetIDQ™ version Oxygen software (Biocrates Life Science AG).

### Statistical analysis

Metabolomic data from EMC patients were compared with data from the ToMMo cohort. Samples from the ToMMo cohort were selected from samples previously measured in the ToMMo project and matched to the EMC group for age, gender, and body mass index (BMI) [23, 24]. To normalize differences in measurements between different batches, we measure four gQC samples per batch. Normalization between batches was corrected for each metabolite by the ratio of the median value of each batch to the median value of all batches. After normalization, no differences were found in metabolite concentrations of gQC between the ToMMo cohort and the EMC group. Multivariate, principal component (PCA), and orthogonal partial least squares-discriminant (OPLS-DA) analyses were performed using MetaboAnalyst 5.0. The two-sided *p* values and false discovery rate (FDR) were calculated using the Wilcoxon rank-sum test with Shapiro–Wilk using GraphPad Prism v8 (GraphPad Software Inc., San Diego, CA, USA). Analysis of the association between risk of recurrence and metabolites was performed by defining low-risk patients as 0, moderate-risk patients as 1, and high-risk patients as 2. A survival curve analysis was performed for metabolites correlated with the risk of recurrence by dividing the metabolite concentrations into two groups, above and below the median, and performing a log-rank test. Differences were considered statistically significant at *p* < 0.05.

## Results

### Sample information and data normalization

Targeted metabolomics was used to analyze plasma samples from 142 patients with EMC. The resulting metabolic profile was compared with that of the ToMMo cohort (control group, age- and BMI-matched, *n* = 154), generated in the same manner as the EMC data. The characteristics of the two groups are summarized in Table 1. From a total of 628 metabolites analyzed, 419 metabolites were analyzed whereas the remaining 209 were detected in less than 80% of the samples and were excluded from further analysis. The mean values and fold change for the EMC and cohort groups for 628 metabolites are shown in Table S1.

**Table 1:** Characteristics of patients with EMC and healthy controls

	**Patients with EMC**	**Healthy controls**	***p* value**
Total (*n*)	142	154	
Age (years, mean ± SD)	59.29 ± 11.67	59.03 ± 12.31	0.51
BMI (kg/m^2^, mean ± SD)	24.55 ± 4.97	23.71 ± 3.33	0.23
FIGO stage, *n* (%)			
I	108 (76.06)		
II	8 (5.63)		
III	12 (8.45)		
IV	14 (9.86)		
Histopathological type, *n* (%)			
Endometrioid	118 (83.09)		
Serous	9 (6.34)		
Clear	4 (2.82)		
Others	11 (7.75)		
Recurrence risk, *n* (%)			
Low-risk	64 (45.07)		
Moderate-risk	35 (24.65)		
High-risk	42 (29.58)		
Unknown	1 (0.70)		

### Comparison of metabolite profiles of patients with EMC and healthy controls

**Fig. 1:**
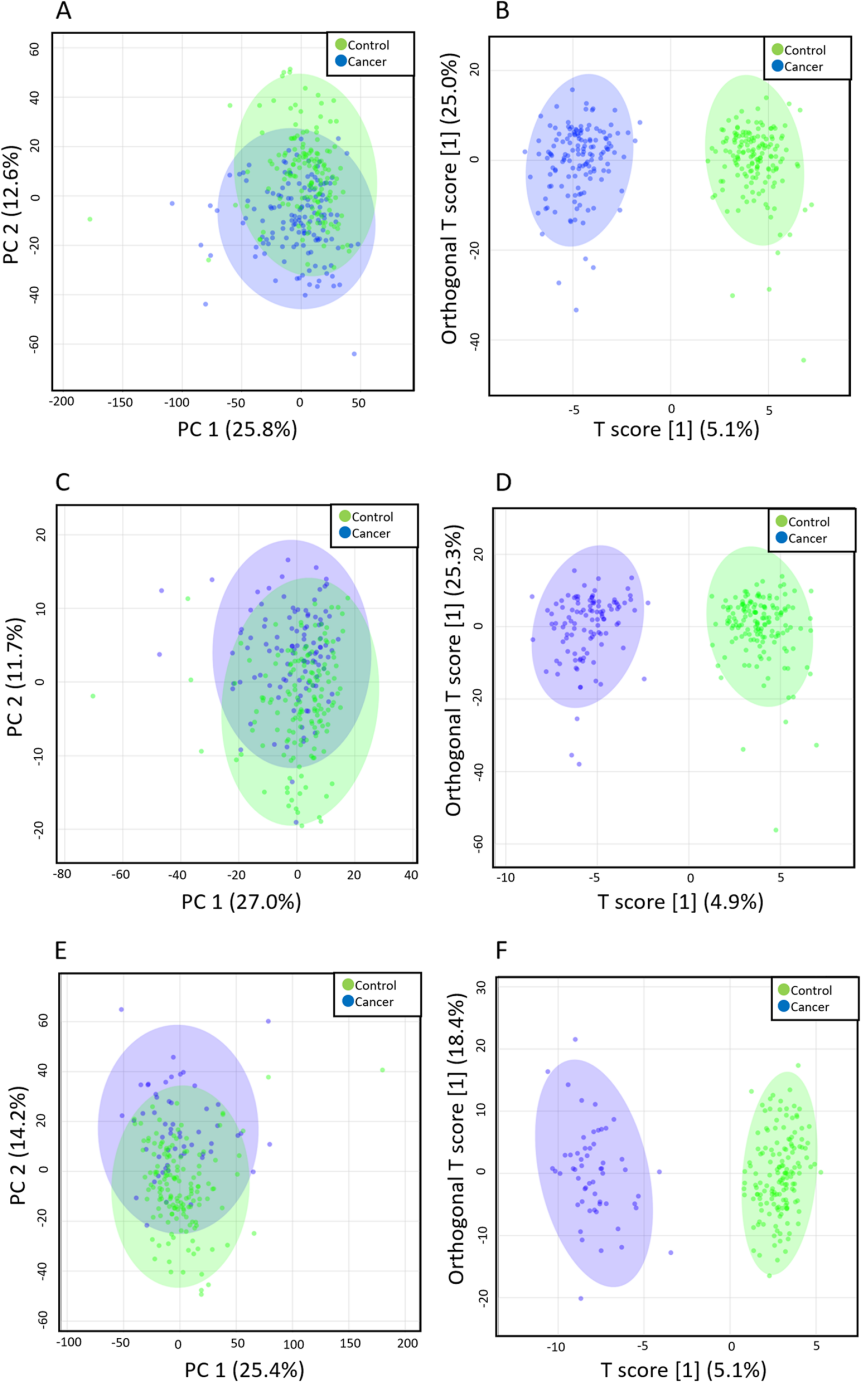
Multivariate analysis of plasma metabolites of patients with EMC (*n* = 142) and healthy controls (*n* = 154). **A** PCA separation of metabolomes of patients with EMC (blue) and healthy controls (green). **B** OPLS-DA separation of metabolomes of patients with EMC (blue) and healthy controls (green). **C** Multivariate analysis of plasma metabolites of patients with stage IA EMC (*n* = 85) and healthy controls (*n* = 154). PCA separation of metabolomes of patients with EMC (blue) and healthy controls (green). **D** OPLS-DA separation of metabolomes of patients with stage IA EMC (blue) and healthy controls (green). **E** Multivariate analysis of plasma metabolites of patients with stage IB-IV EMC (*n* = 57) and healthy controls (*n* = 154). PCA separation of metabolomes of patients with EMC (blue) and healthy controls (green). **F** OPLS-DA separation of metabolomes of patients with stage IB–IV EMC (blue) and healthy controls (green). Each point in the plot corresponds to one plasma sample

PCA revealed a minor whereas OPLS-DA a strong separation in the metabolite profiles between patients with EMC and healthy controls (Fig. 1A, B). Figure 1B illustrates the distinct metabolite profiles of patients with EMC and healthy controls. OPLS-DA creates a discrimination model based on group information. The differences in the levels of each metabolite were also evaluated and the results revealed that the levels of 111 metabolites were significantly increased while those of 148 metabolites significantly decreased in patients with EMC compared with those in healthy controls (Tables S2, S3).

Subsequently, we compared patients with different EMC stages to healthy controls. The PCAs and OPLS-DA of stage IA patients and healthy controls are shown in Fig. 1C and D, whereas those of stage IB–IV patients and healthy controls are shown in Fig. 1E and F, respectively. In both cases, PCA could provide a slight, whereas OPLS-DA a significant separation between the two groups.

**Fig. 2:**
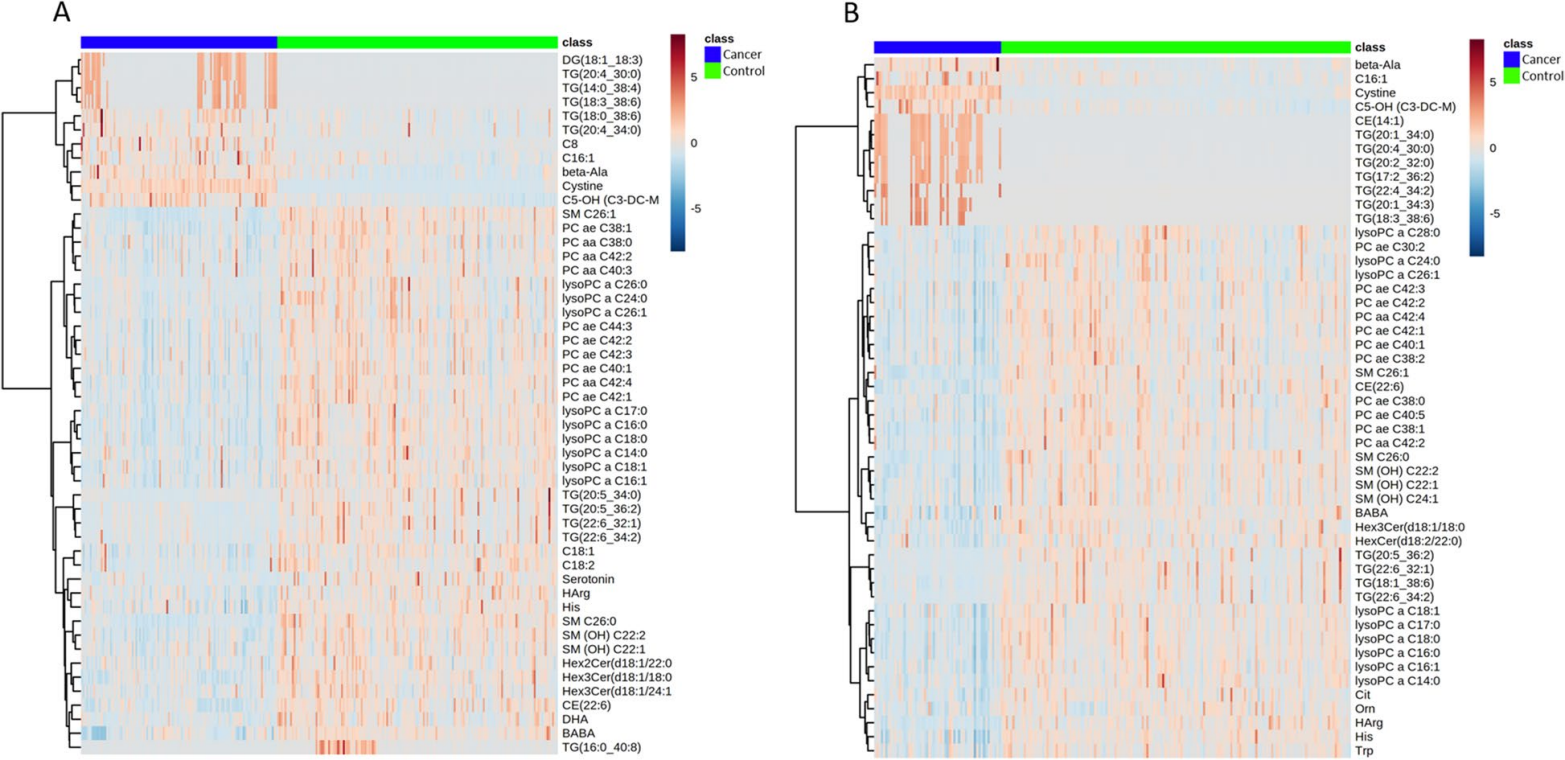
Hierarchical clustering of plasma metabolites of healthy controls and patients with **A** stage IA EMC and **B** stage IB–IV EMC. Rows represent the concentration of each metabolite with a distinct metabolic pattern in patients with EMC and healthy controls. Blue and red bars indicate decreased and increased levels in patients with EMC, respectively, relative to healthy controls. The dendrogram on the left was codirected based on the metabolite concentration profiles

A heatmap of the top 50 metabolites with significantly altered levels in patients with stage IA EMC compared with those in healthy controls is shown in Fig. 2A. A significant increase in the levels of diglyceride (DG) (18:1_18:3), five triglycerides (TGs), acylcarnitine C5–OH (C3–DC–M), C8, C16:1, beta-alanine (Ala), and cystine, and a significant decrease in the levels of four sphingomyelins (SMs), 10 phosphatidylcholines (PCs), nine lysophosphatidylcholines (LysoPCs), five TGs, three hexosylceramides (HexCers), C18:1, C18:2, serotonin, homoarginine (HArg), histidine (His), cholesteryl ester (CE) (22:6), docosahexaenoic acid (DHA), and beta-aminobutyric acid (BABA) was observed. Furthermore, the heatmap of the top 50 metabolites with significantly altered levels in patients with stage IB–IV EMC compared with those in healthy controls is shown in Fig. 2B. A significant increase in the levels of beta-Ala, cysteine, C5–OH (C3–DC–M), C16:1, CE (14:1), and seven TGs were observed as well as a significant decrease in the levels of nine LysoPCs, 11 PCs, five SMs, four TGs, trihexosylceramide (Hex3Cer) (d18:1/18:0), HexCer (d18:2/22:0), CE (22:6), BABA, citrulline (Cit), ornithine (Orn), HArg, His, and tryptophan (Trp).

**Fig. 3:**
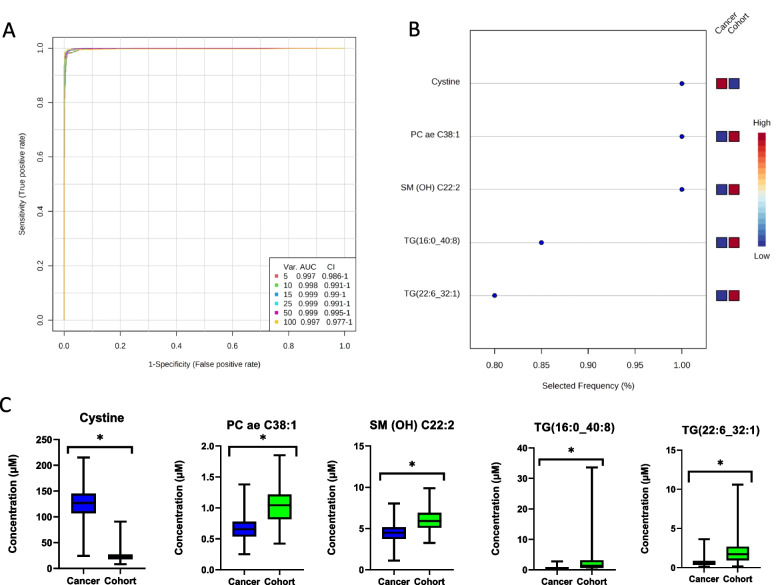
**A** Receiver operating characteristic curve showing the true vs. the false positive rate for a model based on the top 5 to 100 metabolites, used for evaluating the sensitivity and specificity of each metabolite for EMC. The vertical axis of the plot shows sensitivity (true positive rate) and the horizontal axis shows 1-specificity (false positive rate). **B** Metabolites with significant top 5 contributions in the ROC curve. Blue and red bars indicate decreased and increased levels in patients with EMC, respectively, relative to healthy controls. **C** Box plots of metabolites with the top five largest contributions in the ROC curve. *P < 0.05 compared with cohort

An exploratory analysis based on a multivariate receiver operating characteristic curve was performed to assess the sensitivity and specificity of these metabolites (Fig. 3A). The area under the curve for the top five metabolites which included cystine, SM C26:0, SM C26:1, PC C38:1, and C5–OH (C3–DC–M) was 0.997 (95% confidence intervals ranging between 0.986 and 1) (Fig. 3B).

### Identification of metabolites correlated with recurrence risk and stage

**Fig. 4:**
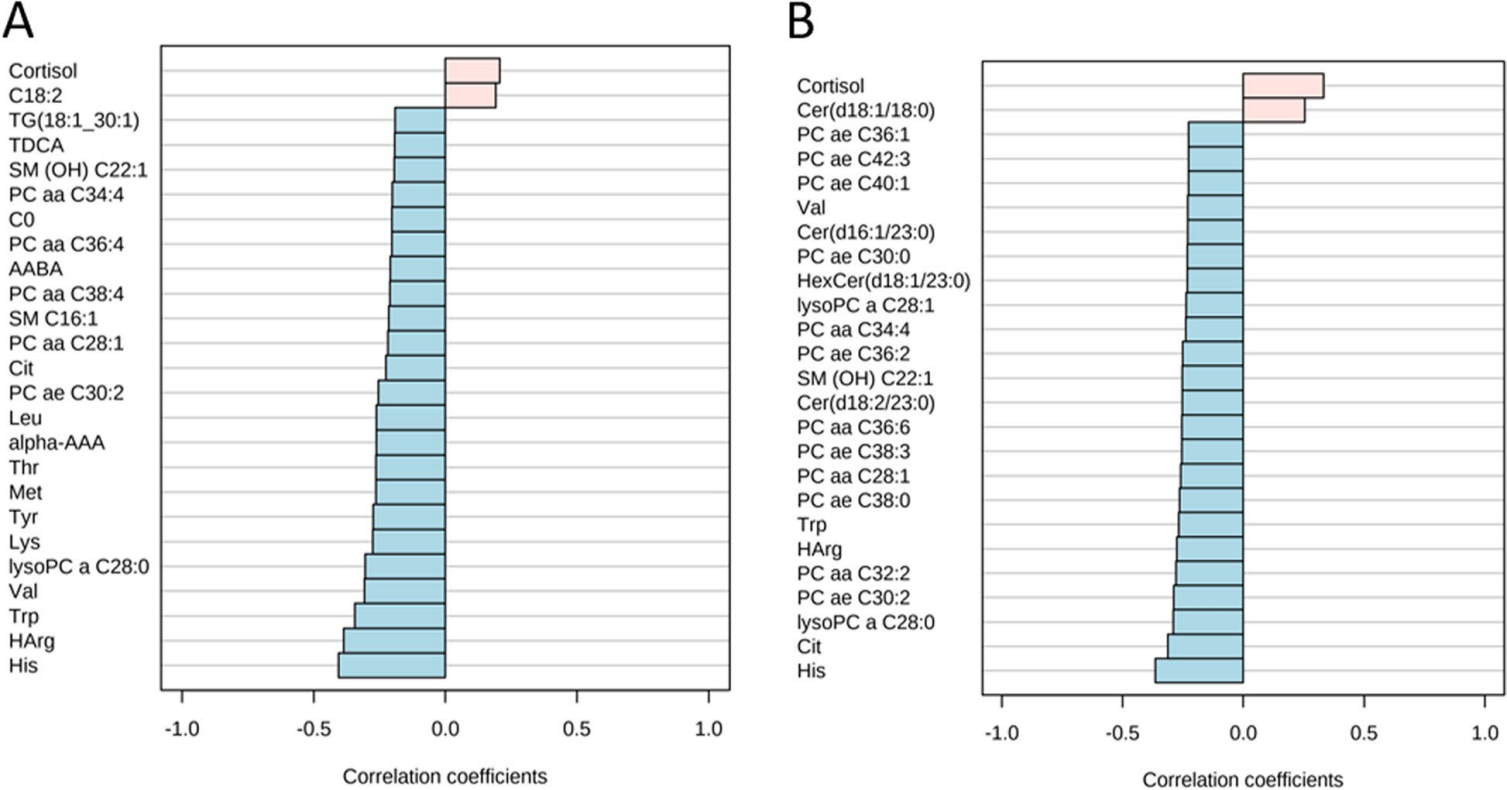
**A** Correlation analysis between metabolite concentrations and risk of recurrence of EMCs. Red and blue bars indicate metabolites that increase or decrease in correlation with the risk of recurrence, respectively. **B** Correlation analysis between metabolite concentrations and stage of EMCs. The red and blue bars indicate metabolites that increase or decrease in correlation with the stage of EMC, respectively

**Fig. 5:**
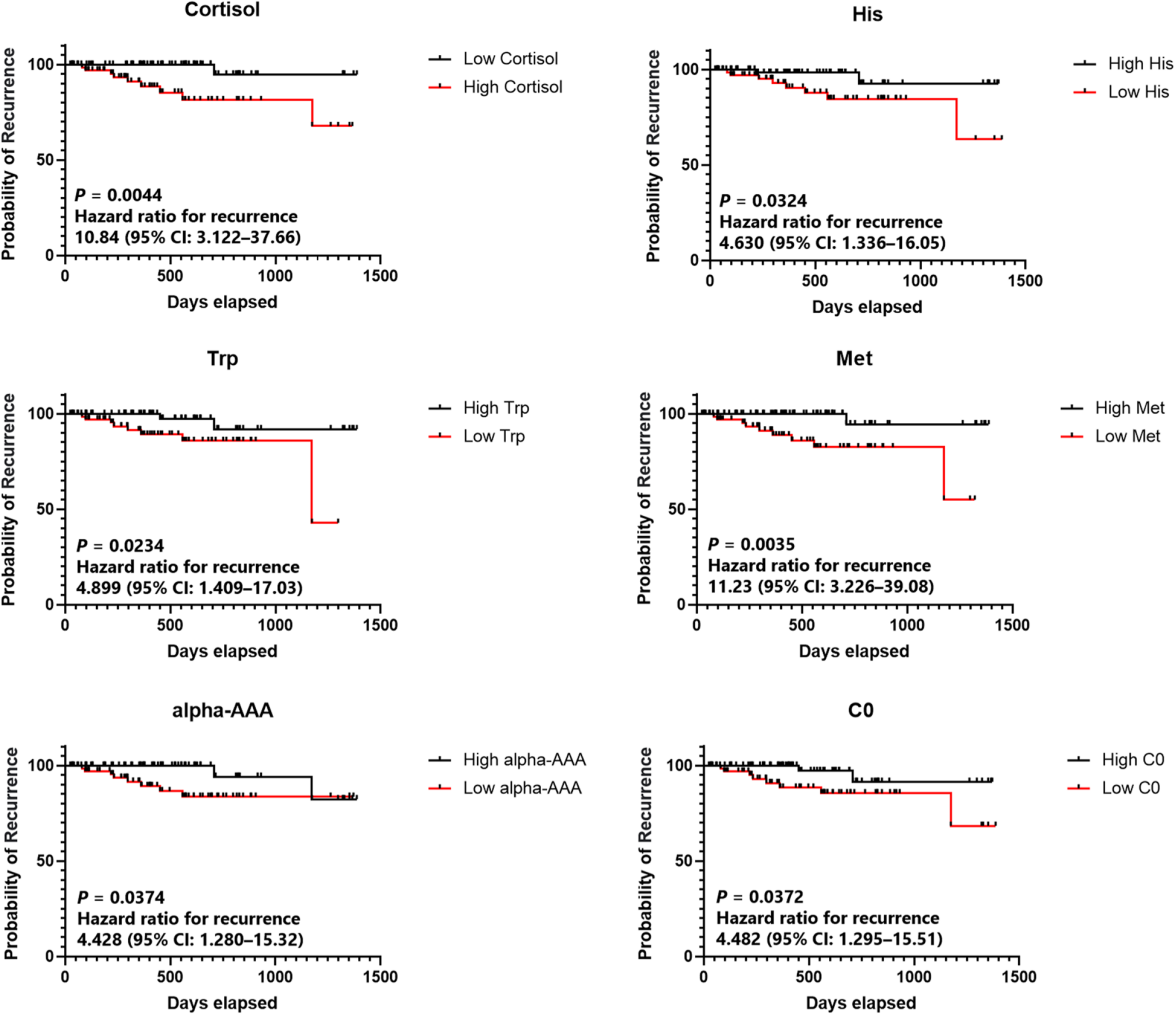
Metabolites associated with relapse in patients with EMC. The Kaplan–Meier survival analysis divided the patients into two groups according to the median metabolite concentration and compared the days of recurrence using the log-rank test. The black line shows group 1, the red line shows group 2, the vertical axis shows the probability of recurrence, and the horizontal axis shows days of elapse

Correlation analysis to investigate whether metabolite changes in EMCs were related to recurrence risk or stage was conducted. The risk of recurrence was categorized into three groups: low-risk, intermediate-risk, and high-risk, whereas the stage was categorized into four groups (stages 1–4). The top 25 metabolites correlated with the risk of recurrence are shown in Fig. 4A. In the high-risk group, only cortisol and C18:2 blood levels were positively correlated with the recurrence risk, whereas the other metabolites, mainly amino acids, were negatively correlated with the risk of recurrence. Similarly, among the 25 metabolites, cortisol and ceramide (Cer) (d18:1/18:0) were positively correlated with the stage, whereas the other metabolites, mainly PCs, exhibited less concentrations as the stage increased (Fig. 4B). Survival curve analysis was then performed on the 25 metabolites that were found to be correlated with the risk of recurrence, in order to verify whether they are related to actual EMC recurrence. The results showed significant differences in six metabolites: cortisol, His, Trp, methionine (Met), alpha-amino adipic acid (alpha-AAA), and carnitine (C0) (Fig. 5). Hazard ratios for relapse were 10.84 (95% CI 3.122–37.66), 4.630 (1.336–16.05), 4.899 (1.409–17.03), 11.23 (3.226–39.08), 4.428 (1.280–15.32), and 4.482 (1.295–15.51), respectively. Kaplan–Meier curves for the remaining 19 metabolites are shown in Supplementary Fig. 1.

## Discussion

Sensitive biomarkers useful for early diagnosis, disease progression, and prognosis of EMC are still unclear. In this study, a comparison of metabolite profiles using plasma metabolome analysis of 142 EMC and 154 healthy control samples revealed that the metabolic profiles of EMC patients were significantly elevated for 111 and significantly decreased for 148 metabolites compared to healthy controls. These metabolites are potential candidates as biomarkers for early diagnosis of EMC. Similar results were observed when comparing patients with stage IA and stage IB–IV EMC with healthy controls. In specifically, LysoPC and TGs containing unsaturated fatty acids levels were found to be reduced in patients with stage IA EMC, also suggesting that these metabolic profiles could be used as diagnostic markers of early-stage EMC. In contrast, in patients with IB and more advanced EMC stages, a decrease in amino acids such as His, Trp, and lipids was observed, with changes correlated with stage and risk of recurrence, probably associated with the progression of EMC. In particular, six metabolites (cortisol, His, Trp, Met, alpha-AAA, and C0) were found to be significantly involved in EMC relapse, suggesting that they are useful predictive markers of EMC relapse.

LysoPC has been reported to be implicated in several cancer pathologies. Metabolic enzymes phospholipases A_1_ and A_2_ convert PC to LysoPC which is subsequently metabolized to lysophosphatidic acid (LPA) by lysophospholipase D (LPD), whose expression is increased in cancer cells [25]. LPA is involved in cancer cell survival, growth, and metastasis via various LPA receptors [26]. The observed marked decrease in plasma LysoPC and PC concentrations in patients with EMC may be due to increased LPA production in the EMC. In a previous study, we reported a similar marked decrease in LysoPC and PC levels in the plasma of patients with ovarian and cervical cancers [15, 16]. Increased phospholipid metabolism is commonly reported in gynecological cancers and constitutes a useful biomarker for monitoring the pathogenesis of these diseases.

Elevated levels of several acylcarnitines in patients with EMC, including C5–OH (C3–DC–M), C8, and C16:1 were also detected. Acylcarnitines transport fatty acids to the mitochondrial membrane to be metabolized through β-oxidation and are common in organisms [27]. Acylcarnitines during cancer metabolism are responsible for supplying fatty acids to cancer cells and may regulate energy production [28]. They have also been reported to be associated with EMC, as suggested by a study by Knific et al. reporting that the ratio of C16 to phosphatidylcholine PC ae C40:1 is an important EMC biomarker [29]. The authors also reported that the ratio of medium- to short-chain ACs was significantly reduced in patients with EMC. In accordance with our results, Kozar et al. reported elevated C14:0, C14:1, and C16:1 levels in the serum of patients with EMC [30].

Sphingolipids play several roles in the etiology and treatment of various cancer types. Ceramide is a complex lipid synthesized from sphingomyelin or palmitoyl-CoA and serine and plays a central role in sphingolipid metabolism [31, 32]. Furthermore, ceramide is converted to sphingosine-1-phosphate (S1P), which promotes tumor growth and survival [33]. In the present study, plasma long-chain SM and HexCer levels are decreased in patients with EMC which is supported by the results of Knific et al. who reported decreased SMOH C14:1 and SMOH C24:1 in the plasma of patients with EMC [29]. The enhanced synthetic pathway of S1P promotes cancer cell growth because it promotes cancer cell proliferation, and the increase in SM and HexCer could be the result of cancer cells requiring sphingolipids to facilitate the synthesis of cellular membranes.

TGs are usually stored in adipocytes and peripheral tissues as a major source of energy in the body, and the levels of TG and CE in organisms are regulated by lecithin-cholesterol acyltransferase (LCAT) [34, 35]. In ovarian cancer, the release of fatty acids from TGs is inhibited, while TGs have been reported to be involved in cancer cell invasion and metastasis [36, 37]. In the present study, we found increased plasma TG levels in patients with EMC which is consistent with our previous results on epithelial ovarian cancer which was associated with increased plasma TG levels [16]. Cheng et al. performed a lipidome analysis of serum from patients with EMC and found a trend towards increased TG levels [38]. In general, patients with a higher body mass index (BMI) tend to have higher triglyceride levels which are elevated in EMC patients, even when compared to similar BMI controls, suggesting that TG regulation is important in EMC [39]. Interestingly, total TGs increased in the patients in the EMC group, whereas decreases in TG (20:5_34:0), TG (20:5_36:2), TG (22:6_32:1), and TG (22:6_34:2) including polyunsaturated fatty acids (PUFA) were observed in the same group. TGs are also a source of free fatty acids (FFAs). Increased biosynthesis of and altered FFA levels are associated with cancer. In cancer cells, PUFAs and their downstream metabolites regulate various processes, such as cell signaling, neurotransmission, cell growth and protection, and inflammation. Cancer cells require unsaturated fatty acids, which affect cancer cell growth and survival via stearoyl-CoA desaturase-1 upregulation [40]. The decrease in the typical DHA and eicosapentaenoic acid (EPA), PUFAs, in the group of patients with EMC suggests that the regulation of PUFAs in the blood of patients with EMC is very important for their treatment.

To explore the metabolites associated with disease prognosis in patients with EMC (Fig. 5), a correlation analysis of the metabolite concentrations with the risk of recurrence and stage was performed. The results identified cortisol as a metabolite that is positively correlated with both the risk of recurrence and stage. Cortisol is a steroid hormone secreted by the adrenal cortex, which is essential for humans and has a wide range of effects such as promoting gluconeogenesis, protein metabolism, lipid metabolism, anti-inflammation, and immunosuppression [41]. Cancer development triggers various inflammatory responses, and the immunosuppressive effects of cortisol may promote immune evasion and acquisition of additional oncogenic mutations in cancer [42]. Furthermore, weight gain and insulin resistance, which have obesity-promoting and blood-glucose-elevating effects, are also associated with an increased risk of various malignancies [43, 44]. Susanna et al. conducted a Mendelian randomized analysis of the relationship between plasma cortisol and cancer risk and reported that increased plasma cortisol levels may increase the risk of EMC [45]. This is consistent with our results, suggesting that high blood cortisol levels may be a marker of EMC progression and prognosis.

Amino acids and their derivatives play important roles in many biochemical processes in vivo and are regulated by metabolic reprogramming in cancer cells, such as the Warburg effect [46]. In the present study, cystine was significantly increased and His, Trp, Cit, Orn, and serotonin were significantly decreased in the plasma of patients with EMC compared to healthy controls. Cystine is a source of cysteine, which is transported into cells via the xCT antiporter and is involved in the synthesis of the antioxidant glutathione [47]. Sendo et al. reported a reduced expression of the cysteine transporter xCT, in an endometrial cancer cell line [47]. Elevated cystine levels in the plasma of patients with EMC result from a reduced cellular uptake of cysteine, suggesting that glutathione metabolism may be affected in EMC. Both Cit and Orn are intermediates of the urea biochemical cycle, which controls ammonia and nitric oxide (NO) metabolism regulation through the production of Arg. NO, which plays a major role in oxidative stress and other stress conditions such as inflammation and cancer, is produced by macrophages as a result of the inflammatory stimulation of inducible nitric oxide synthase [48]. Jacopo et al. reported lower serum urea concentrations in patients with EMC, indicating a metabolic shift towards suppression of the urea cycle pathway and increased NO production [49].

The role of Trp metabolism in the prognosis of epithelial ovarian and cervical cancer was elucidated previously, as indicated by the significantly reduced plasma His and Trp levels recorded in these cancers [15, 16]. In the tumor microenvironment, indoleamine-2,3-dioxygenase (IDO) catabolizes Trp to produce kynurenine, which suppresses the immune response of T cells facilitating tumor survival [50, 51]. Similar reductions in Trp and its downstream serotonin have been observed in the present study in the plasma of patients with EMC. Furthermore, His and Trp levels were negatively correlated with the risk of recurrence and stage, rendering them potential predictive markers of disease progression and prognosis of EMC.

## Conclusion

In summary, the plasma metabolome analysis of patients with EMC and comparisons with healthy controls identified specific metabolites associated with the pathogenesis of EMC. These metabolites were also correlated with the risk of recurrence and disease stage. However, the detailed temporal changes and histological differences remain unknown and require further validation using larger sample sizes. Overall, the results of the present study suggest that analysis of changes in the plasma metabolome profile could be used for the early diagnosis, disease assessment, and monitoring of the course of EMC treatment.

### Supplementary Information

**Additional file 1: Table S1. **List of all metabolites measured in this study.** Table S2. **Significantly increased metabolites in the plasma of patients with endometrial cancer compared to healthy controls. **Table S3.** Significantly decreased metabolites in the plasma of patients with endometrial cancer compared to healthy controls.

**Additional file 2: Fig. S1. **Kaplan-Meier survival analysis of metabolites correlated with risk of recurrence in EMC patients. The Kaplan-Meier survival analysis divided the patients into two groups according to the median metabolite concentration and compared the days of recurrence using the log-rank test. The black line shows Group 1, the red line shows Group 2, the vertical axis shows probability of recurrence and the horizontal axis shows days of elapse. Shown for 19 metabolites that are not significant between the two groups.

## Data Availability

The data that support the findings of this study are available on request from the corresponding author. The data are not publicly available due to privacy or ethical restrictions. In addition, the data about identifiable human research participants cannot be openly shared.

## Abbreviations

alpha-AAA: Alpha-aminoadipic acid
AC: Acylcarnitine
Ala: Alanine
BABA: Beta-aminobutyric acid
BMI: Body mass index
C0: Carnitine
Cer: Ceramide
CE: Cholesteryl ester
Cit: Citrulline
CN-H: Copy number high
CN-L: Copy number low
DG: Diglyceride
DHA: Docosahexaenoic acid
EMC: Endometrial cancer
EPA: Eicosapentaenoic acid
FFA: Free fatty acid
gQC: Global quality control
HArg: Homoarginine
HexCer: Hexosylceramides
Hex3Cer: Trihexosylceramide
His: Histidine
JSOG: Japanese Society of Obstetrics and Gynecology
LCAT: Lecithin-cholesterol acyltransferase
LPA: Lysophosphatidic acid
LPD: Lysophospholipase D
LysoPC: Lysophosphatidylcholines
Met: Methionine
MSI: Microsatellite instability
NO: Nitric oxide
OPLS-DA: Orthogonal partial least squares-discriminant analysis
Orn: Ornithine
PC: Phosphatidylcholine
PCA: Principal component analysis
POLE: Polymerase ε
PUFA: Polyunsaturated fatty acids
SM: Sphingomyelin
S1P: Sphingosine-1-phosphate
TG: Triglyceride
Trp: Tryptophan
UHPLC-MS/MS: Ultra-high-performance liquid chromatography-tandem mass spectrometry
