# Supplementary material for: Identification of predictive biomarkers for endometrial cancer diagnosis and treatment response monitoring using plasma metabolome profiling
Source: Cancer Metab. 2023 Oct 11;11:16. doi: 10.1186/s40170-023-00317-z (PMC10568780; doi:10.1186/s40170-023-00317-z)
Supplement: Supplementary file 1 — Additional file 1: Table S1. List of all metabolites measured in this study. Table S2. Significantly increased metabolites in the plasma of patients with endometrial cancer compared to healthy controls. Table S3. Significantly decreased metabolites in the plasma of patients with endometrial cancer compared to healthy controls. [file 40170_2023_317_MOESM1_ESM.docx]

**Table S1**

List of all metabolites measured in this study

| Metabolite | Class | Cancer mean (µM) | Cancer SD | Cohort mean (µM) | Cohort SD | Fold change (Cancer/Cohort) |
| --- | --- | --- | --- | --- | --- | --- |
| 1-Met-His | Amino acid related | 1.80 | 0.57 | 2.10 | 2.00 | 1.17 |
| 3-IAA | Indoles and derivatives | 0.15 | 0.10 | 0.17 | 0.13 | 1.16 |
| 3-IPA | Indoles and derivatives | 1.64 | 7.21 | 1.38 | 5.12 | 0.84 |
| 3-Met-His | Amino acid related | 2.72 | 3.82 | 2.63 | 3.50 | 0.97 |
| 5-AVA | Amino acid related | 0.05 | 0.03 | 0.06 | 0.03 | 1.06 |
| AA | Fatty acids | 21.79 | 10.10 | 20.81 | 10.67 | 0.95 |
| AABA | Amino acid related | 12.22 | 3.53 | 12.83 | 4.61 | 1.05 |
| AbsAcid | Hormones and related | 0.00 | 0.00 | 0.00 | 0.00 | 1.28 |
| AconAcid | Carboxylic acids | 11.77 | 3.12 | 10.27 | 2.62 | 0.87 |
| Ac-Orn | Amino acid related | N.D. | N.D. | N.D. | N.D. | N.D. |
| ADMA | Amino acid related | 0.50 | 0.07 | 0.46 | 0.08 | 0.93 |
| Ala | Amino acids | 370.40 | 84.22 | 380.79 | 117.43 | 1.03 |
| alpha-AAA | Amino acid related | 0.59 | 0.27 | 0.67 | 0.33 | 1.13 |
| Anserine | Amino acid related | 0.00 | 0.01 | 0.01 | 0.05 | 3.54 |
| Arg | Amino acids | 71.64 | 22.48 | 78.92 | 24.69 | 1.10 |
| Asn | Amino acids | 48.34 | 9.50 | 47.71 | 14.07 | 0.99 |
| Asp | Amino acids | 7.98 | 1.85 | 7.13 | 3.01 | 0.89 |
| BABA | Amino acid related | 0.23 | 0.04 | 0.16 | 0.08 | 0.70 |
| beta-Ala | Biogenic amines | 0.05 | 0.04 | 0.11 | 0.08 | 2.41 |
| Betaine | Amino acid related | 12.00 | 4.29 | 12.09 | 3.78 | 1.01 |
| C0 | Acylcarnitines | 51.89 | 10.36 | 47.44 | 10.22 | 0.91 |
| C10 | Acylcarnitines | 0.24 | 0.10 | 0.22 | 0.14 | 0.92 |
| C10:1 | Acylcarnitines | N.D. | N.D. | N.D. | N.D. | N.D. |
| C10:2 | Acylcarnitines | 0.14 | 0.03 | 0.09 | 0.01 | 0.65 |
| C12 | Acylcarnitines | 0.07 | 0.03 | 0.07 | 0.04 | 0.98 |
| C12:1 | Acylcarnitines | N.D. | N.D. | N.D. | N.D. | N.D. |
| C12-DC | Acylcarnitines | N.D. | N.D. | N.D. | N.D. | N.D. |
| C14 | Acylcarnitines | 0.04 | 0.02 | 0.05 | 0.02 | 1.15 |
| C14:1 | Acylcarnitines | 0.08 | 0.04 | 0.07 | 0.03 | 0.84 |
| C14:1-OH | Acylcarnitines | 0.04 | 0.02 | 0.04 | 0.02 | 1.12 |
| C14:2 | Acylcarnitines | 0.14 | 0.12 | 0.12 | 0.13 | 0.86 |
| C14:2-OH | Acylcarnitines | 0.03 | 0.03 | 0.03 | 0.01 | 0.94 |
| C16 | Acylcarnitines | 0.13 | 0.03 | 0.13 | 0.04 | 0.95 |
| C16:1 | Acylcarnitines | 0.07 | 0.02 | 0.10 | 0.06 | 1.46 |
| C16:1-OH | Acylcarnitines | 0.02 | 0.02 | 0.02 | 0.01 | 1.07 |
| C16:2 | Acylcarnitines | 0.02 | 0.01 | 0.02 | 0.01 | 1.06 |
| C16:2-OH | Acylcarnitines | 0.07 | 0.03 | 0.08 | 0.10 | 1.04 |
| C16-OH | Acylcarnitines | 0.03 | 0.02 | N.D. | N.D. | N.D. |
| C18 | Acylcarnitines | 0.04 | 0.01 | 0.04 | 0.01 | 0.93 |
| C18:1 | Acylcarnitines | 0.20 | 0.05 | 0.16 | 0.05 | 0.78 |
| C18:1-OH | Acylcarnitines | 0.10 | 0.06 | 0.08 | 0.03 | 0.77 |
| C18:2 | Acylcarnitines | 0.10 | 0.04 | 0.07 | 0.03 | 0.71 |
| C2 | Acylcarnitines | 8.37 | 3.10 | 7.13 | 3.03 | 0.85 |
| C3 | Acylcarnitines | 0.36 | 0.14 | 0.37 | 0.16 | 1.02 |
| C3:1 | Acylcarnitines | 0.05 | 0.04 | 0.01 | 0.00 | 0.32 |
| C3-DC (C4-OH) | Acylcarnitines | 0.08 | 0.05 | 0.08 | 0.06 | 1.04 |
| C3-OH | Acylcarnitines | 0.15 | 0.07 | 0.06 | 0.01 | 0.37 |
| C4 | Acylcarnitines | 0.17 | 0.07 | 0.18 | 0.07 | 1.08 |
| C4:1 | Acylcarnitines | 0.04 | 0.02 | 0.08 | 0.02 | 2.04 |
| c4-OH-Pro | Amino acid related | N.D. | N.D. | N.D. | N.D. | N.D. |
| C5 | Acylcarnitines | 0.12 | 0.67 | 0.11 | 0.57 | 0.88 |
| C5:1 | Acylcarnitines | N.D. | N.D. | 0.01 | 0.00 | N.D. |
| C5:1-DC | Acylcarnitines | 0.03 | 0.03 | 0.03 | 0.03 | 0.84 |
| C5-DC (C6-OH) | Acylcarnitines | 0.05 | 0.01 | 0.05 | 0.01 | 1.02 |
| C5-M-DC | Acylcarnitines | 0.13 | 0.06 | 0.14 | 0.12 | 1.08 |
| C5-OH (C3-DC-M) | Acylcarnitines | 0.03 | 0.01 | 0.05 | 0.02 | 1.69 |
| C6 (C4:1-DC) | Acylcarnitines | 0.10 | 0.04 | 0.11 | 0.07 | 1.07 |
| C6:1 | Acylcarnitines | 0.03 | 0.01 | 0.04 | 0.02 | 1.24 |
| C7-DC | Acylcarnitines | 0.04 | 0.03 | N.D. | N.D. | N.D. |
| C8 | Acylcarnitines | 0.20 | 0.06 | 0.52 | 0.53 | 2.61 |
| C9 | Acylcarnitines | 0.04 | 0.01 | 0.04 | 0.01 | 1.12 |
| CA | Bile acids | 0.08 | 0.21 | 0.07 | 0.17 | 0.95 |
| Carnosine | Amino acid related | N.D. | N.D. | N.D. | N.D. | N.D. |
| CDCA | Bile acids | 0.19 | 0.32 | 0.26 | 0.56 | 1.42 |
| CE(14:0) | Cholesteryl esters | 9.14 | 2.73 | 7.05 | 5.67 | 0.77 |
| CE(14:1) | Cholesteryl esters | 0.22 | 0.08 | 0.43 | 0.40 | 1.95 |
| CE(15:0) | Cholesteryl esters | 2.21 | 0.51 | 2.14 | 0.62 | 0.97 |
| CE(15:1) | Cholesteryl esters | 0.63 | 0.19 | 0.72 | 0.32 | 1.14 |
| CE(16:0) | Cholesteryl esters | 40.97 | 10.48 | 47.99 | 35.87 | 1.17 |
| CE(16:1) | Cholesteryl esters | 14.22 | 6.48 | 16.54 | 7.56 | 1.16 |
| CE(17:0) | Cholesteryl esters | 3.65 | 1.99 | 3.77 | 2.80 | 1.03 |
| CE(17:1) | Cholesteryl esters | 1.53 | 0.69 | 1.96 | 1.25 | 1.28 |
| CE(18:0) | Cholesteryl esters | 5.06 | 1.68 | 6.12 | 3.66 | 1.21 |
| CE(18:1) | Cholesteryl esters | 78.03 | 19.31 | 68.82 | 21.42 | 0.88 |
| CE(18:2) | Cholesteryl esters | 371.47 | 97.73 | 352.45 | 116.51 | 0.95 |
| CE(18:3) | Cholesteryl esters | 20.38 | 7.04 | 18.02 | 8.12 | 0.88 |
| CE(20:0) | Cholesteryl esters | 11.22 | 6.94 | 6.94 | 5.50 | 0.62 |
| CE(20:1) | Cholesteryl esters | 1.22 | 0.55 | 7.19 | 5.66 | 5.89 |
| CE(20:3) | Cholesteryl esters | 6.39 | 2.00 | 6.12 | 1.78 | 0.96 |
| CE(20:4) | Cholesteryl esters | 69.60 | 21.83 | 67.85 | 24.18 | 0.97 |
| CE(20:5) | Cholesteryl esters | 83.85 | 59.63 | 59.09 | 58.00 | 0.70 |
| CE(22:0) | Cholesteryl esters | 0.96 | N.D. | 0.93 | 0.28 | 0.97 |
| CE(22:1) | Cholesteryl esters | 0.17 | 0.08 | 0.83 | 0.34 | 4.97 |
| CE(22:2) | Cholesteryl esters | 0.20 | 0.14 | 0.52 | 0.37 | 2.66 |
| CE(22:5) | Cholesteryl esters | 2.07 | 0.70 | 2.46 | 1.40 | 1.19 |
| CE(22:6) | Cholesteryl esters | 46.59 | 16.24 | 26.37 | 12.38 | 0.57 |
| Cer(d16:1/18:0) | Ceramides | 0.11 | 0.05 | 0.10 | 0.05 | 0.86 |
| Cer(d16:1/20:0) | Ceramides | 0.14 | 0.05 | 0.12 | 0.06 | 0.88 |
| Cer(d16:1/22:0) | Ceramides | 0.22 | 0.08 | 0.19 | 0.07 | 0.88 |
| Cer(d16:1/23:0) | Ceramides | 0.09 | 0.04 | 0.08 | 0.03 | 0.88 |
| Cer(d16:1/24:0) | Ceramides | 0.15 | 0.06 | 0.12 | 0.05 | 0.77 |
| Cer(d18:0/18:0(OH)) | Ceramides | 0.48 | 0.31 | 0.46 | 0.23 | 0.95 |
| Cer(d18:0/18:0) | Ceramides | 0.02 | 0.01 | 0.02 | 0.01 | 0.87 |
| Cer(d18:0/20:0) | Ceramides | 0.21 | 0.10 | 0.22 | 0.15 | 1.05 |
| Cer(d18:0/22:0) | Ceramides | 0.22 | 0.08 | 0.23 | 0.17 | 1.07 |
| Cer(d18:0/24:0) | Ceramides | 0.31 | 0.10 | 0.42 | 0.27 | 1.37 |
| Cer(d18:0/24:1) | Ceramides | 0.35 | 0.10 | 0.48 | 0.31 | 1.36 |
| Cer(d18:0/26:1(OH)) | Ceramides | 1.93 | 4.56 | 0.72 | 0.49 | 0.37 |
| Cer(d18:0/26:1) | Ceramides | 0.05 | 0.03 | 0.04 | 0.03 | 0.88 |
| Cer(d18:1/14:0) | Ceramides | 0.06 | 0.03 | 0.06 | 0.03 | 0.86 |
| Cer(d18:1/16:0) | Ceramides | 0.47 | 0.09 | 0.48 | 0.12 | 1.01 |
| Cer(d18:1/18:0(OH)) | Ceramides | 0.33 | 0.12 | 0.26 | 0.16 | 0.79 |
| Cer(d18:1/18:0) | Ceramides | 0.14 | 0.04 | 0.15 | 0.04 | 1.07 |
| Cer(d18:1/18:1) | Ceramides | 0.03 | 0.01 | 0.02 | 0.01 | 0.81 |
| Cer(d18:1/20:0(OH)) | Ceramides | 0.51 | 0.20 | 0.32 | 0.24 | 0.63 |
| Cer(d18:1/20:0) | Ceramides | 0.11 | 0.03 | 0.12 | 0.03 | 1.06 |
| Cer(d18:1/22:0) | Ceramides | 0.68 | 0.18 | 0.67 | 0.18 | 0.99 |
| Cer(d18:1/23:0) | Ceramides | 0.73 | 0.22 | 0.69 | 0.20 | 0.95 |
| Cer(d18:1/24:0) | Ceramides | 1.75 | 0.45 | 1.59 | 0.47 | 0.91 |
| Cer(d18:1/24:1) | Ceramides | 1.12 | 0.21 | 1.09 | 0.25 | 0.97 |
| Cer(d18:1/25:0) | Ceramides | 0.33 | 0.09 | 0.31 | 0.07 | 0.94 |
| Cer(d18:1/26:0) | Ceramides | 0.05 | 0.02 | 0.05 | 0.03 | 1.07 |
| Cer(d18:1/26:1) | Ceramides | 0.03 | 0.01 | 0.03 | 0.01 | 0.79 |
| Cer(d18:2/14:0) | Ceramides | 0.01 | 0.00 | 0.01 | 0.01 | 0.88 |
| Cer(d18:2/16:0) | Ceramides | 0.11 | 0.02 | 0.11 | 0.04 | 1.00 |
| Cer(d18:2/18:0) | Ceramides | 0.07 | 0.02 | 0.08 | 0.03 | 1.03 |
| Cer(d18:2/18:1) | Ceramides | 0.01 | 0.01 | 0.02 | 0.02 | 1.43 |
| Cer(d18:2/20:0) | Ceramides | 0.08 | 0.02 | 0.09 | 0.04 | 1.07 |
| Cer(d18:2/22:0) | Ceramides | 0.25 | 0.08 | 0.22 | 0.06 | 0.89 |
| Cer(d18:2/23:0) | Ceramides | 0.11 | 0.04 | 0.10 | 0.03 | 0.86 |
| Cer(d18:2/24:0) | Ceramides | 0.39 | 0.12 | 0.33 | 0.11 | 0.84 |
| Cer(d18:2/24:1) | Ceramides | 0.24 | 0.06 | 0.22 | 0.06 | 0.92 |
| Choline | Vitamins and cofactors | 12.06 | 2.74 | 10.55 | 3.27 | 0.87 |
| Cit | Amino acid related | 35.56 | 8.95 | 29.05 | 7.82 | 0.82 |
| Cortisol | Hormones and related | 0.20 | 0.07 | 0.23 | 0.09 | 1.15 |
| Cortisone | Hormones and related | 0.06 | 0.01 | 0.07 | 0.01 | 1.14 |
| Creatinine | Amino acid related | 64.25 | 16.69 | 58.79 | 26.24 | 0.92 |
| Cys | Amino acids | 154.21 | 29.50 | 151.50 | 34.49 | 0.98 |
| Cystine | Amino acid related | 24.53 | 12.42 | 125.43 | 32.84 | 5.11 |
| DCA | Bile acids | 0.28 | 0.41 | 0.23 | 0.23 | 0.82 |
| DG(14:0_14:0) | Diglycerides | N.D. | N.D. | 6.28 | 2.50 | N.D. |
| DG(14:0_18:1) | Diglycerides | 2.66 | N.D. | 1.67 | 1.29 | 0.63 |
| DG(14:0_18:2) | Diglycerides | N.D. | N.D. | N.D. | N.D. | N.D. |
| DG(14:0_20:0) | Diglycerides | 0.50 | 0.28 | 0.57 | 0.10 | 1.15 |
| DG(14:1_18:1) | Diglycerides | 0.07 | 0.03 | 0.09 | 0.04 | 1.27 |
| DG(14:1_20:2) | Diglycerides | 2.10 | 2.32 | 0.78 | 0.37 | 0.37 |
| DG(16:0_16:0) | Diglycerides | N.D. | N.D. | N.D. | N.D. | N.D. |
| DG(16:0_16:1) | Diglycerides | 0.20 | 0.25 | 0.55 | 0.62 | 2.71 |
| DG(16:0_18:1) | Diglycerides | 1.47 | 1.11 | 1.82 | 1.15 | 1.24 |
| DG(16:0_18:2) | Diglycerides | 0.86 | 0.52 | 1.11 | 0.65 | 1.29 |
| DG(16:0_20:0) | Diglycerides | 0.26 | 0.11 | 0.64 | 1.16 | 2.42 |
| DG(16:0_20:3) | Diglycerides | 0.09 | 0.05 | 0.12 | 0.06 | 1.42 |
| DG(16:0_20:4) | Diglycerides | 0.84 | 0.71 | 0.45 | 0.14 | 0.53 |
| DG(16:1_18:0) | Diglycerides | 0.12 | 0.10 | N.D. | N.D. | N.D. |
| DG(16:1_18:1) | Diglycerides | 8.03 | 7.04 | 15.06 | 5.11 | 1.88 |
| DG(16:1_18:2) | Diglycerides | 0.36 | 0.19 | 0.36 | 0.18 | 1.00 |
| DG(16:1_20:0) | Diglycerides | 0.11 | 0.09 | 0.15 | 0.09 | 1.38 |
| DG(17:0_17:1) | Diglycerides | 0.65 | 0.98 | 0.38 | 0.18 | 0.58 |
| DG(17:0_18:1) | Diglycerides | 0.36 | 0.18 | 0.48 | 0.29 | 1.32 |
| DG(18:0_20:0) | Diglycerides | 3.59 | 1.73 | 3.32 | 0.77 | 0.92 |
| DG(18:0_20:4) | Diglycerides | 0.49 | 0.31 | 0.29 | 0.17 | 0.59 |
| DG(18:1_18:1) | Diglycerides | 1.56 | 0.79 | 1.88 | 0.88 | 1.21 |
| DG(18:1_18:2) | Diglycerides | 3.53 | 1.58 | 4.21 | 1.89 | 1.19 |
| DG(18:1_18:3) | Diglycerides | 0.31 | 0.15 | 0.43 | 0.46 | 1.37 |
| DG(18:1_18:4) | Diglycerides | 0.21 | 0.17 | 0.21 | 0.19 | 0.98 |
| DG(18:1_20:0) | Diglycerides | 0.25 | 0.17 | 0.27 | 0.14 | 1.07 |
| DG(18:1_20:1) | Diglycerides | 0.06 | 0.03 | 0.05 | 0.03 | 0.78 |
| DG(18:1_20:2) | Diglycerides | 0.15 | 0.07 | 0.16 | 0.10 | 1.04 |
| DG(18:1_20:3) | Diglycerides | 0.07 | 0.02 | 0.04 | 0.02 | 0.64 |
| DG(18:1_20:4) | Diglycerides | 0.55 | 0.26 | 0.61 | 0.34 | 1.11 |
| DG(18:1_22:5) | Diglycerides | 0.09 | 0.05 | 0.13 | 0.05 | 1.44 |
| DG(18:1_22:6) | Diglycerides | 2.88 | 1.98 | 3.16 | 1.07 | 1.10 |
| DG(18:2_18:2) | Diglycerides | 1.82 | 0.87 | 2.07 | 1.08 | 1.14 |
| DG(18:2_18:3) | Diglycerides | 0.79 | 0.43 | 0.90 | 0.92 | 1.13 |
| DG(18:2_18:4) | Diglycerides | 0.13 | 0.03 | 0.14 | 0.06 | 1.06 |
| DG(18:2_20:0) | Diglycerides | 0.15 | 0.07 | 0.17 | 0.10 | 1.16 |
| DG(18:2_20:4) | Diglycerides | 0.23 | 0.13 | 0.30 | 0.11 | 1.33 |
| DG(18:3_18:3) | Diglycerides | 1.11 | 0.74 | 1.00 | 0.58 | 0.90 |
| DG(18:3_20:2) | Diglycerides | 0.62 | 0.38 | 0.42 | 0.24 | 0.67 |
| DG(21:0_22:6) | Diglycerides | 0.17 | 0.03 | 0.19 | 0.09 | 1.10 |
| DG(22:1_22:2) | Diglycerides | 0.11 | 0.09 | 0.11 | 0.08 | 0.99 |
| DG-O(14:0_18:2) | Diglycerides | N.D. | N.D. | 10.90 | 2.33 | N.D. |
| DG-O(16:0_18:1) | Diglycerides | 0.06 | 0.04 | 0.04 | 0.02 | 0.72 |
| DG-O(18:2_18:2) | Diglycerides | 0.04 | 0.02 | 0.04 | 0.02 | 1.09 |
| DHA | Fatty acids | 101.15 | 61.58 | 60.56 | 44.61 | 0.60 |
| DHEAS | Hormones and related | 2.41 | 1.42 | 2.13 | 1.31 | 0.88 |
| DiCA(12:0) | Carboxylic acids | 0.64 | N.D. | 0.69 | 0.13 | 1.07 |
| DiCA(14:0) | Carboxylic acids | 0.05 | 0.02 | 0.05 | 0.02 | 1.02 |
| DOPA | Amino acid related | 2.98 | N.D. | 0.58 | 0.77 | 0.19 |
| Dopamine | Biogenic amines | N.D. | N.D. | N.D. | N.D. | N.D. |
| EPA | Fatty acids | 9.15 | 7.33 | 5.78 | 6.47 | 0.63 |
| FA(12:0) | Fatty acids | 16.72 | 5.20 | 20.01 | 5.20 | 1.20 |
| FA(14:0) | Fatty acids | 519.75 | 123.50 | 347.00 | 147.95 | 0.67 |
| FA(16:0) | Fatty acids | N.D. | N.D. | 518.60 | 11.22 | N.D. |
| FA(18:0) | Fatty acids | N.D. | N.D. | 903.00 | N.D. | N.D. |
| FA(18:1) | Fatty acids | 152.41 | 62.63 | 127.68 | 58.77 | 0.84 |
| FA(18:2) | Fatty acids | 326.94 | 119.58 | 293.67 | 153.29 | 0.90 |
| FA(20:1) | Fatty acids | 9.94 | 5.95 | 8.43 | 8.00 | 0.85 |
| FA(20:2) | Fatty acids | 0.91 | 0.65 | 0.75 | 0.44 | 0.82 |
| FA(20:3) | Fatty acids | 0.85 | 0.46 | 0.84 | 0.56 | 0.98 |
| GABA | Biogenic amines | 0.17 | 0.03 | 0.15 | 0.04 | 0.90 |
| GCA | Bile acids | 0.20 | 0.47 | 0.22 | 0.29 | 1.13 |
| GCDCA | Bile acids | 0.44 | 0.61 | 0.67 | 0.71 | 1.51 |
| GDCA | Bile acids | 0.24 | 0.37 | 0.23 | 0.26 | 0.94 |
| GLCA | Bile acids | 0.02 | 0.03 | 0.03 | 0.06 | 1.80 |
| GLCAS | Bile acids | 0.10 | 0.11 | 0.11 | 0.15 | 1.05 |
| Gln | Amino acids | 636.98 | 83.33 | 585.17 | 87.00 | 0.92 |
| Glu | Amino acids | 49.34 | 15.84 | 56.81 | 30.96 | 1.15 |
| Gly | Amino acids | 255.79 | 87.51 | 226.01 | 75.13 | 0.88 |
| GUDCA | Bile acids | 0.26 | 0.39 | 0.51 | 1.15 | 1.94 |
| H1 | Carbohydrates and related | 4578.16 | 995.03 | 5067.84 | 1528.58 | 1.11 |
| HArg | Amino acid related | 5.22 | 1.93 | 3.41 | 1.55 | 0.65 |
| HCys | Amino acid related | 3.23 | 1.12 | 2.75 | 1.25 | 0.85 |
| Hex2Cer(d18:1/14:0) | Dihexosylceramides | 0.17 | 0.05 | 0.17 | 0.05 | 1.04 |
| Hex2Cer(d18:1/16:0) | Dihexosylceramides | 2.13 | 0.49 | 2.25 | 0.60 | 1.06 |
| Hex2Cer(d18:1/18:0) | Dihexosylceramides | 0.17 | 0.03 | 0.17 | 0.04 | 1.00 |
| Hex2Cer(d18:1/20:0) | Dihexosylceramides | 0.12 | 0.04 | 0.09 | 0.02 | 0.79 |
| Hex2Cer(d18:1/22:0) | Dihexosylceramides | 0.23 | 0.06 | 0.19 | 0.05 | 0.83 |
| Hex2Cer(d18:1/24:0) | Dihexosylceramides | 0.19 | 0.05 | 0.18 | 0.05 | 0.95 |
| Hex2Cer(d18:1/24:1) | Dihexosylceramides | 0.36 | 0.09 | 0.34 | 0.10 | 0.94 |
| Hex2Cer(d18:1/26:0) | Dihexosylceramides | 0.02 | 0.01 | 0.01 | 0.01 | 0.70 |
| Hex2Cer(d18:1/26:1) | Dihexosylceramides | 0.01 | 0.01 | 0.01 | 0.02 | 0.89 |
| Hex3Cer(d18:1/16:0) | Trihexosylceramides | 1.40 | 0.33 | 1.29 | 0.29 | 0.92 |
| Hex3Cer(d18:1/18:0) | Trihexosylceramides | 0.19 | 0.05 | 0.13 | 0.05 | 0.70 |
| Hex3Cer(d18:1/20:0) | Trihexosylceramides | 0.10 | 0.03 | 0.08 | 0.04 | 0.82 |
| Hex3Cer(d18:1/22:0) | Trihexosylceramides | 0.21 | 0.07 | 0.17 | 0.05 | 0.81 |
| Hex3Cer(d18:1/24:1) | Trihexosylceramides | 0.70 | 0.21 | 0.58 | 0.17 | 0.82 |
| Hex3Cer(d18:1/26:1) | Trihexosylceramides | 0.08 | 0.03 | 0.07 | 0.02 | 0.86 |
| HexCer(d16:1/20:0) | Hexosylceramides | 0.04 | 0.01 | 0.05 | 0.03 | 1.26 |
| HexCer(d16:1/22:0) | Hexosylceramides | 0.05 | 0.01 | 0.05 | 0.03 | 0.99 |
| HexCer(d16:1/24:0) | Hexosylceramides | 0.12 | 0.04 | 0.14 | 0.07 | 1.17 |
| HexCer(d18:1/14:0) | Hexosylceramides | 0.10 | 0.03 | 0.08 | 0.05 | 0.80 |
| HexCer(d18:1/16:0) | Hexosylceramides | 1.08 | 0.22 | 1.16 | 0.30 | 1.07 |
| HexCer(d18:1/18:0) | Hexosylceramides | 0.23 | 0.07 | 0.22 | 0.09 | 0.96 |
| HexCer(d18:1/18:1) | Hexosylceramides | 0.14 | 0.04 | 0.14 | 0.05 | 0.99 |
| HexCer(d18:1/20:0) | Hexosylceramides | 0.37 | 0.11 | 0.40 | 0.23 | 1.10 |
| HexCer(d18:1/22:0) | Hexosylceramides | 3.10 | 0.87 | 2.90 | 0.82 | 0.94 |
| HexCer(d18:1/23:0) | Hexosylceramides | 1.32 | 0.35 | 1.27 | 0.35 | 0.96 |
| HexCer(d18:1/24:0) | Hexosylceramides | 1.62 | 0.45 | 1.51 | 0.42 | 0.93 |
| HexCer(d18:1/24:1) | Hexosylceramides | 4.40 | 0.98 | 4.09 | 1.02 | 0.93 |
| HexCer(d18:1/26:0) | Hexosylceramides | 0.16 | 0.07 | 0.19 | 0.08 | 1.17 |
| HexCer(d18:1/26:1) | Hexosylceramides | 0.26 | 0.06 | 0.31 | 0.12 | 1.16 |
| HexCer(d18:2/16:0) | Hexosylceramides | 0.05 | 0.01 | 0.06 | 0.03 | 1.18 |
| HexCer(d18:2/18:0) | Hexosylceramides | 0.04 | 0.01 | 0.02 | 0.01 | 0.61 |
| HexCer(d18:2/20:0) | Hexosylceramides | 0.04 | 0.01 | 0.05 | 0.03 | 1.23 |
| HexCer(d18:2/22:0) | Hexosylceramides | 0.78 | 0.21 | 0.62 | 0.23 | 0.80 |
| HexCer(d18:2/23:0) | Hexosylceramides | 0.22 | 0.08 | 0.24 | 0.10 | 1.06 |
| HexCer(d18:2/24:0) | Hexosylceramides | 0.94 | 0.27 | 0.85 | 0.27 | 0.90 |
| HipAcid | Carboxylic acids | 1.15 | 1.56 | 1.15 | 1.33 | 1.01 |
| His | Amino acids | 85.00 | 10.11 | 73.33 | 15.73 | 0.86 |
| Histamine | Biogenic amines | N.D. | N.D. | N.D. | N.D. | N.D. |
| Hypoxanthine | Nucleobases and related | 2.62 | 1.47 | 9.46 | 21.68 | 3.61 |
| Ile | Amino acids | 62.28 | 14.78 | 69.64 | 20.68 | 1.12 |
| Indole | Indoles and derivatives | N.D. | N.D. | 7.45 | 0.95 | N.D. |
| Ind-SO4 | Indoles and derivatives | 3.75 | 2.38 | 3.80 | 4.42 | 1.01 |
| Kynurenine | Amino acid related | 1.56 | 0.44 | 1.68 | 0.53 | 1.08 |
| Lac | Carboxylic acids | 2965.34 | 709.03 | 2569.80 | 1865.67 | 0.87 |
| Leu | Amino acids | 107.01 | 19.44 | 105.99 | 27.32 | 0.99 |
| Lys | Amino acids | 188.42 | 31.33 | 180.16 | 44.09 | 0.96 |
| lysoPC a C14:0 | Lysophosphatidylcholines | 1.70 | 0.57 | 1.27 | 0.50 | 0.75 |
| lysoPC a C16:0 | Lysophosphatidylcholines | 119.15 | 25.03 | 86.01 | 23.20 | 0.72 |
| lysoPC a C16:1 | Lysophosphatidylcholines | 3.01 | 0.76 | 2.30 | 0.82 | 0.76 |
| lysoPC a C17:0 | Lysophosphatidylcholines | 1.94 | 0.54 | 1.32 | 0.43 | 0.68 |
| lysoPC a C18:0 | Lysophosphatidylcholines | 38.96 | 9.27 | 26.71 | 8.54 | 0.69 |
| lysoPC a C18:1 | Lysophosphatidylcholines | 22.53 | 5.11 | 17.63 | 6.21 | 0.78 |
| lysoPC a C18:2 | Lysophosphatidylcholines | 30.18 | 9.38 | 27.56 | 11.20 | 0.91 |
| lysoPC a C20:3 | Lysophosphatidylcholines | 1.80 | 0.59 | 1.62 | 0.71 | 0.90 |
| lysoPC a C20:4 | Lysophosphatidylcholines | 5.95 | 1.46 | 5.19 | 1.72 | 0.87 |
| lysoPC a C24:0 | Lysophosphatidylcholines | 0.28 | 0.09 | 0.22 | 0.05 | 0.77 |
| lysoPC a C26:0 | Lysophosphatidylcholines | 0.38 | 0.15 | 0.28 | 0.07 | 0.74 |
| lysoPC a C26:1 | Lysophosphatidylcholines | 0.54 | 0.17 | 0.41 | 0.10 | 0.77 |
| lysoPC a C28:0 | Lysophosphatidylcholines | 0.63 | 0.17 | 0.51 | 0.12 | 0.81 |
| lysoPC a C28:1 | Lysophosphatidylcholines | 0.63 | 0.17 | 0.55 | 0.15 | 0.86 |
| Met | Amino acids | 23.95 | 4.19 | 22.01 | 5.69 | 0.92 |
| Met-SO | Amino acid related | 0.72 | 0.27 | 0.70 | 0.34 | 0.97 |
| Nitro-Tyr | Amino acid related | N.D. | N.D. | N.D. | N.D. | N.D. |
| OH-GlutAcid | Carboxylic acids | N.D. | N.D. | N.D. | N.D. | N.D. |
| Orn | Amino acid related | 93.52 | 23.39 | 76.06 | 28.88 | 0.81 |
| PAG | Amino acid related | 0.01 | 0.00 | 0.01 | N.D. | 1.08 |
| PC aa C24:0 | Phosphatidylcholines | 0.15 | 0.08 | 0.11 | 0.05 | 0.71 |
| PC aa C26:0 | Phosphatidylcholines | 2.05 | 0.41 | 1.90 | 0.26 | 0.93 |
| PC aa C28:1 | Phosphatidylcholines | 3.93 | 0.97 | 3.48 | 0.87 | 0.89 |
| PC aa C30:0 | Phosphatidylcholines | 3.89 | 1.18 | 3.74 | 1.37 | 0.96 |
| PC aa C32:0 | Phosphatidylcholines | 11.57 | 2.39 | 11.35 | 2.39 | 0.98 |
| PC aa C32:1 | Phosphatidylcholines | 11.13 | 5.70 | 12.77 | 6.78 | 1.15 |
| PC aa C32:2 | Phosphatidylcholines | 2.88 | 0.93 | 2.70 | 1.05 | 0.94 |
| PC aa C32:3 | Phosphatidylcholines | 0.50 | 0.10 | 0.46 | 0.11 | 0.91 |
| PC aa C34:1 | Phosphatidylcholines | 173.97 | 46.28 | 175.36 | 47.28 | 1.01 |
| PC aa C34:2 | Phosphatidylcholines | 297.47 | 67.03 | 299.44 | 71.19 | 1.01 |
| PC aa C34:3 | Phosphatidylcholines | 12.07 | 3.45 | 11.86 | 4.11 | 0.98 |
| PC aa C34:4 | Phosphatidylcholines | 1.22 | 0.39 | 1.10 | 0.45 | 0.90 |
| PC aa C36:0 | Phosphatidylcholines | 2.03 | 0.67 | 1.65 | 0.63 | 0.81 |
| PC aa C36:1 | Phosphatidylcholines | 40.84 | 11.00 | 37.82 | 11.26 | 0.93 |
| PC aa C36:2 | Phosphatidylcholines | 187.76 | 41.83 | 175.28 | 44.09 | 0.93 |
| PC aa C36:3 | Phosphatidylcholines | 83.96 | 21.84 | 79.50 | 22.53 | 0.95 |
| PC aa C36:4 | Phosphatidylcholines | 118.49 | 28.77 | 120.14 | 28.76 | 1.01 |
| PC aa C36:5 | Phosphatidylcholines | 41.55 | 23.97 | 33.90 | 18.23 | 0.82 |
| PC aa C36:6 | Phosphatidylcholines | 1.44 | 0.59 | 1.09 | 0.45 | 0.76 |
| PC aa C38:0 | Phosphatidylcholines | 3.50 | 0.81 | 2.92 | 0.76 | 0.84 |
| PC aa C38:3 | Phosphatidylcholines | 34.16 | 8.39 | 31.09 | 7.90 | 0.91 |
| PC aa C38:4 | Phosphatidylcholines | 69.18 | 17.87 | 67.38 | 15.75 | 0.97 |
| PC aa C38:5 | Phosphatidylcholines | 52.12 | 17.42 | 44.35 | 12.94 | 0.85 |
| PC aa C38:6 | Phosphatidylcholines | 121.92 | 33.99 | 102.65 | 27.89 | 0.84 |
| PC aa C40:1 | Phosphatidylcholines | 0.63 | 0.17 | 0.56 | 0.14 | 0.90 |
| PC aa C40:2 | Phosphatidylcholines | 0.37 | 0.18 | 0.28 | 0.12 | 0.75 |
| PC aa C40:3 | Phosphatidylcholines | 0.60 | 0.20 | 0.46 | 0.17 | 0.77 |
| PC aa C40:4 | Phosphatidylcholines | 1.74 | 0.46 | 1.58 | 0.48 | 0.91 |
| PC aa C40:5 | Phosphatidylcholines | 7.37 | 2.47 | 6.35 | 2.04 | 0.86 |
| PC aa C40:6 | Phosphatidylcholines | 40.48 | 13.65 | 33.06 | 9.56 | 0.82 |
| PC aa C42:0 | Phosphatidylcholines | 0.56 | 0.12 | 0.52 | 0.17 | 0.93 |
| PC aa C42:1 | Phosphatidylcholines | 0.32 | 0.06 | 0.29 | 0.08 | 0.91 |
| PC aa C42:2 | Phosphatidylcholines | 0.27 | 0.07 | 0.21 | 0.06 | 0.80 |
| PC aa C42:4 | Phosphatidylcholines | 0.15 | 0.03 | 0.12 | 0.02 | 0.80 |
| PC aa C42:5 | Phosphatidylcholines | 0.36 | 0.13 | 0.28 | 0.10 | 0.78 |
| PC aa C42:6 | Phosphatidylcholines | 0.60 | 0.28 | 0.44 | 0.21 | 0.73 |
| PC ae C30:0 | Phosphatidylcholines | 0.35 | 0.10 | 0.33 | 0.09 | 0.92 |
| PC ae C30:1 | Phosphatidylcholines | 2.34 | 0.71 | 2.08 | 0.71 | 0.89 |
| PC ae C30:2 | Phosphatidylcholines | 0.16 | 0.04 | 0.14 | 0.03 | 0.84 |
| PC ae C32:1 | Phosphatidylcholines | 2.28 | 0.48 | 2.11 | 0.50 | 0.93 |
| PC ae C32:2 | Phosphatidylcholines | 0.81 | 0.19 | 0.69 | 0.19 | 0.86 |
| PC ae C34:0 | Phosphatidylcholines | 1.12 | 0.30 | 1.04 | 0.30 | 0.93 |
| PC ae C34:1 | Phosphatidylcholines | 6.69 | 1.19 | 6.48 | 1.38 | 0.97 |
| PC ae C34:2 | Phosphatidylcholines | 8.20 | 1.85 | 7.47 | 1.85 | 0.91 |
| PC ae C34:3 | Phosphatidylcholines | 6.00 | 1.62 | 5.22 | 1.67 | 0.87 |
| PC ae C36:0 | Phosphatidylcholines | 1.00 | 0.34 | 0.90 | 0.25 | 0.90 |
| PC ae C36:1 | Phosphatidylcholines | 6.29 | 1.35 | 5.69 | 1.39 | 0.90 |
| PC ae C36:2 | Phosphatidylcholines | 9.29 | 2.10 | 8.79 | 2.14 | 0.95 |
| PC ae C36:3 | Phosphatidylcholines | 4.99 | 1.09 | 4.47 | 1.14 | 0.90 |
| PC ae C36:4 | Phosphatidylcholines | 10.41 | 2.32 | 9.99 | 2.43 | 0.96 |
| PC ae C36:5 | Phosphatidylcholines | 7.91 | 1.67 | 7.20 | 2.01 | 0.91 |
| PC ae C38:0 | Phosphatidylcholines | 2.51 | 0.75 | 1.98 | 0.61 | 0.79 |
| PC ae C38:1 | Phosphatidylcholines | 1.05 | 0.29 | 0.67 | 0.20 | 0.64 |
| PC ae C38:2 | Phosphatidylcholines | 1.91 | 0.46 | 1.63 | 0.39 | 0.85 |
| PC ae C38:3 | Phosphatidylcholines | 2.57 | 0.49 | 2.36 | 0.52 | 0.92 |
| PC ae C38:4 | Phosphatidylcholines | 6.49 | 1.41 | 6.40 | 1.51 | 0.99 |
| PC ae C38:5 | Phosphatidylcholines | 11.54 | 1.97 | 10.73 | 2.17 | 0.93 |
| PC ae C38:6 | Phosphatidylcholines | 7.47 | 1.78 | 6.34 | 1.74 | 0.85 |
| PC ae C40:1 | Phosphatidylcholines | 1.37 | 0.31 | 1.12 | 0.31 | 0.82 |
| PC ae C40:2 | Phosphatidylcholines | 1.62 | 0.39 | 1.44 | 0.33 | 0.89 |
| PC ae C40:3 | Phosphatidylcholines | 0.83 | 0.14 | 0.75 | 0.14 | 0.91 |
| PC ae C40:4 | Phosphatidylcholines | 1.41 | 0.26 | 1.30 | 0.29 | 0.92 |
| PC ae C40:5 | Phosphatidylcholines | 2.65 | 0.51 | 2.28 | 0.50 | 0.86 |
| PC ae C40:6 | Phosphatidylcholines | 4.55 | 1.04 | 3.91 | 1.00 | 0.86 |
| PC ae C42:0 | Phosphatidylcholines | 0.59 | 0.17 | 0.66 | 0.13 | 1.11 |
| PC ae C42:1 | Phosphatidylcholines | 0.31 | 0.07 | 0.25 | 0.05 | 0.79 |
| PC ae C42:2 | Phosphatidylcholines | 0.47 | 0.10 | 0.38 | 0.09 | 0.81 |
| PC ae C42:3 | Phosphatidylcholines | 0.74 | 0.13 | 0.64 | 0.14 | 0.86 |
| PC ae C42:4 | Phosphatidylcholines | 0.55 | 0.12 | 0.53 | 0.15 | 0.97 |
| PC ae C42:5 | Phosphatidylcholines | 1.38 | 0.22 | 1.32 | 0.30 | 0.96 |
| PC ae C44:3 | Phosphatidylcholines | 0.14 | 0.03 | 0.12 | 0.02 | 0.86 |
| PC ae C44:4 | Phosphatidylcholines | 0.22 | 0.05 | 0.22 | 0.06 | 0.98 |
| PC ae C44:5 | Phosphatidylcholines | 0.95 | 0.25 | 1.02 | 0.32 | 1.07 |
| PC ae C44:6 | Phosphatidylcholines | 0.93 | 0.19 | 0.92 | 0.26 | 1.00 |
| p-Cresol-SO4 | Cresols | 41.67 | 33.25 | 36.56 | 30.45 | 0.88 |
| PEA | Biogenic amines | N.D. | N.D. | N.D. | N.D. | N.D. |
| Phe | Amino acids | 59.40 | 8.77 | 59.52 | 12.42 | 1.00 |
| PheAlaBetaine | Amino acid related | 0.01 | 0.01 | 0.02 | 0.02 | 2.04 |
| Pro | Amino acids | 138.65 | 39.33 | 152.23 | 47.10 | 1.10 |
| ProBetaine | Amino acid related | 3.12 | 3.10 | 4.10 | 4.20 | 1.31 |
| Putrescine | Biogenic amines | 0.12 | 0.08 | 0.11 | 0.08 | 0.91 |
| Sarcosine | Amino acid related | 1.43 | 1.59 | 2.90 | 3.74 | 2.04 |
| SDMA | Amino acid related | 0.51 | 0.14 | 0.53 | 0.20 | 1.05 |
| Ser | Amino acids | 122.73 | 24.33 | 110.76 | 26.03 | 0.90 |
| Serotonin | Biogenic amines | 0.27 | 0.20 | 0.14 | 0.11 | 0.51 |
| SM (OH) C14:1 | Sphingomyelins | 3.59 | 0.91 | 3.31 | 0.81 | 0.92 |
| SM (OH) C16:1 | Sphingomyelins | 2.05 | 0.51 | 1.92 | 0.45 | 0.94 |
| SM (OH) C22:1 | Sphingomyelins | 7.02 | 1.60 | 5.78 | 1.46 | 0.82 |
| SM (OH) C22:2 | Sphingomyelins | 6.04 | 1.32 | 4.48 | 1.04 | 0.74 |
| SM (OH) C24:1 | Sphingomyelins | 0.67 | 0.16 | 0.56 | 0.12 | 0.84 |
| SM C16:0 | Sphingomyelins | 86.19 | 16.15 | 79.67 | 14.24 | 0.92 |
| SM C16:1 | Sphingomyelins | 12.22 | 2.40 | 11.52 | 2.30 | 0.94 |
| SM C18:0 | Sphingomyelins | 16.18 | 3.75 | 16.38 | 3.79 | 1.01 |
| SM C18:1 | Sphingomyelins | 7.60 | 1.88 | 7.54 | 1.85 | 0.99 |
| SM C20:2 | Sphingomyelins | 0.22 | 0.07 | 0.24 | 0.07 | 1.11 |
| SM C24:0 | Sphingomyelins | 12.73 | 2.61 | 11.58 | 2.73 | 0.91 |
| SM C24:1 | Sphingomyelins | 33.05 | 6.66 | 30.25 | 6.02 | 0.92 |
| SM C26:0 | Sphingomyelins | 0.09 | 0.03 | 0.05 | 0.01 | 0.56 |
| SM C26:1 | Sphingomyelins | 0.29 | 0.10 | 0.13 | 0.07 | 0.46 |
| Spermidine | Biogenic amines | 0.26 | N.D. | 0.17 | 0.07 | 0.66 |
| Spermine | Biogenic amines | N.D. | N.D. | 0.57 | 0.11 | N.D. |
| Suc | Carboxylic acids | 20.90 | 1.49 | 23.38 | 2.31 | 1.12 |
| t4-OH-Pro | Amino acid related | 8.48 | 3.88 | 8.85 | 5.05 | 1.04 |
| Taurine | Amino acid related | 74.61 | 20.95 | 61.33 | 20.95 | 0.82 |
| TCA | Bile acids | 0.05 | 0.13 | 0.06 | 0.08 | 1.05 |
| TCDCA | Bile acids | 0.09 | 0.15 | 0.13 | 0.15 | 1.42 |
| TDCA | Bile acids | 0.05 | 0.09 | 0.05 | 0.06 | 0.91 |
| TG(14:0_32:2) | Triglycerides | 0.39 | 0.58 | 0.65 | 0.62 | 1.64 |
| TG(14:0_34:0) | Triglycerides | 0.57 | 1.08 | 0.65 | 0.54 | 1.14 |
| TG(14:0_34:1) | Triglycerides | 3.47 | 5.07 | 4.82 | 5.58 | 1.39 |
| TG(14:0_34:2) | Triglycerides | 2.37 | 2.57 | 3.26 | 3.24 | 1.37 |
| TG(14:0_34:3) | Triglycerides | 0.66 | 0.56 | 0.88 | 0.73 | 1.33 |
| TG(14:0_35:1) | Triglycerides | 0.12 | 0.17 | 0.25 | 0.42 | 2.09 |
| TG(14:0_35:2) | Triglycerides | 0.16 | 0.16 | 0.36 | 0.43 | 2.25 |
| TG(14:0_36:1) | Triglycerides | 0.85 | 0.90 | 1.27 | 1.19 | 1.50 |
| TG(14:0_36:2) | Triglycerides | 4.74 | 3.31 | 6.15 | 4.85 | 1.30 |
| TG(14:0_36:3) | Triglycerides | 4.59 | 2.65 | 5.61 | 4.09 | 1.22 |
| TG(14:0_36:4) | Triglycerides | 1.58 | 0.93 | 1.87 | 1.40 | 1.18 |
| TG(14:0_38:4) | Triglycerides | 0.12 | 0.07 | 0.18 | 0.20 | 1.57 |
| TG(14:0_38:5) | Triglycerides | 0.17 | 0.12 | 0.25 | 0.31 | 1.51 |
| TG(14:0_40:5) | Triglycerides | 0.24 | 0.13 | 0.21 | 0.07 | 0.84 |
| TG(16:0_28:1) | Triglycerides | 0.65 | 1.40 | 0.72 | 1.31 | 1.10 |
| TG(16:0_28:2) | Triglycerides | 0.21 | 0.33 | 0.37 | 0.42 | 1.76 |
| TG(16:0_30:2) | Triglycerides | 0.54 | 1.09 | 0.77 | 1.19 | 1.41 |
| TG(16:0_32:0) | Triglycerides | 5.33 | 13.39 | 5.45 | 5.74 | 1.02 |
| TG(16:0_32:1) | Triglycerides | 6.44 | 12.13 | 8.85 | 10.36 | 1.37 |
| TG(16:0_32:2) | Triglycerides | 2.89 | 3.84 | 3.93 | 4.19 | 1.36 |
| TG(16:0_32:3) | Triglycerides | 0.54 | 0.60 | 0.76 | 0.77 | 1.41 |
| TG(16:0_33:1) | Triglycerides | 1.02 | 1.75 | 0.98 | 0.87 | 0.97 |
| TG(16:0_33:2) | Triglycerides | 0.46 | 0.69 | 0.51 | 0.39 | 1.11 |
| TG(16:0_34:0) | Triglycerides | 3.24 | 6.44 | 5.14 | 3.99 | 1.58 |
| TG(16:0_34:1) | Triglycerides | 28.05 | 42.43 | 41.07 | 30.99 | 1.46 |
| TG(16:0_34:2) | Triglycerides | 25.27 | 26.97 | 35.61 | 25.97 | 1.41 |
| TG(16:0_34:3) | Triglycerides | 8.32 | 6.70 | 10.89 | 7.29 | 1.31 |
| TG(16:0_34:4) | Triglycerides | 1.00 | 0.88 | 1.17 | 0.90 | 1.17 |
| TG(16:0_35:1) | Triglycerides | 0.58 | 0.83 | 0.86 | 0.61 | 1.48 |
| TG(16:0_35:2) | Triglycerides | 0.98 | 0.86 | 1.41 | 0.95 | 1.43 |
| TG(16:0_35:3) | Triglycerides | 0.49 | 0.33 | 0.62 | 0.36 | 1.28 |
| TG(16:0_36:2) | Triglycerides | 62.32 | 44.03 | 84.55 | 49.51 | 1.36 |
| TG(16:0_36:3) | Triglycerides | 61.48 | 33.62 | 79.11 | 42.43 | 1.29 |
| TG(16:0_36:4) | Triglycerides | 21.61 | 11.52 | 26.46 | 15.15 | 1.22 |
| TG(16:0_36:5) | Triglycerides | 3.39 | 2.17 | 3.84 | 2.49 | 1.13 |
| TG(16:0_36:6) | Triglycerides | 0.52 | 0.45 | 0.41 | 0.29 | 0.80 |
| TG(16:0_37:3) | Triglycerides | 0.23 | 0.16 | 0.31 | 0.16 | 1.36 |
| TG(16:0_38:1) | Triglycerides | 0.26 | 0.26 | 0.28 | 0.17 | 1.07 |
| TG(16:0_38:2) | Triglycerides | 0.83 | 0.63 | 0.97 | 0.59 | 1.17 |
| TG(16:0_38:3) | Triglycerides | 1.05 | 0.63 | 1.35 | 0.74 | 1.28 |
| TG(16:0_38:4) | Triglycerides | 1.16 | 0.76 | 1.40 | 0.80 | 1.21 |
| TG(16:0_38:5) | Triglycerides | 1.53 | 1.07 | 1.79 | 1.03 | 1.17 |
| TG(16:0_38:6) | Triglycerides | 1.65 | 1.30 | 1.66 | 1.07 | 1.01 |
| TG(16:0_38:7) | Triglycerides | 1.15 | 1.53 | 0.43 | 0.28 | 0.38 |
| TG(16:0_40:6) | Triglycerides | 1.31 | 0.74 | 1.39 | 0.70 | 1.06 |
| TG(16:0_40:7) | Triglycerides | 2.39 | 1.79 | 1.54 | 1.02 | 0.64 |
| TG(16:0_40:8) | Triglycerides | 3.76 | 5.76 | 0.59 | 0.42 | 0.16 |
| TG(16:1_28:0) | Triglycerides | 0.55 | 1.20 | 0.26 | 0.47 | 0.48 |
| TG(16:1_30:1) | Triglycerides | 1.68 | 5.37 | 0.60 | 0.80 | 0.36 |
| TG(16:1_32:0) | Triglycerides | 3.10 | 9.64 | 1.51 | 1.86 | 0.49 |
| TG(16:1_32:1) | Triglycerides | 2.97 | 7.17 | 2.33 | 2.44 | 0.79 |
| TG(16:1_32:2) | Triglycerides | 0.68 | 0.79 | 0.76 | 0.60 | 1.13 |
| TG(16:1_33:1) | Triglycerides | 0.37 | 0.50 | 0.38 | 0.25 | 1.04 |
| TG(16:1_34:0) | Triglycerides | 1.36 | 2.28 | 1.63 | 1.37 | 1.20 |
| TG(16:1_34:1) | Triglycerides | 10.51 | 11.48 | 13.94 | 10.85 | 1.33 |
| TG(16:1_34:2) | Triglycerides | 7.52 | 5.67 | 8.96 | 5.58 | 1.19 |
| TG(16:1_34:3) | Triglycerides | 1.76 | 1.11 | 1.80 | 1.06 | 1.02 |
| TG(16:1_36:1) | Triglycerides | 1.52 | 1.25 | 1.74 | 1.07 | 1.15 |
| TG(16:1_36:2) | Triglycerides | 9.45 | 5.04 | 10.70 | 5.45 | 1.13 |
| TG(16:1_36:3) | Triglycerides | 8.39 | 3.85 | 9.15 | 4.58 | 1.09 |
| TG(16:1_36:4) | Triglycerides | 3.17 | 1.53 | 3.28 | 1.79 | 1.03 |
| TG(16:1_36:5) | Triglycerides | 0.62 | 0.33 | 0.59 | 0.32 | 0.95 |
| TG(16:1_38:3) | Triglycerides | 0.19 | 0.10 | 0.19 | 0.08 | 1.02 |
| TG(16:1_38:4) | Triglycerides | 0.25 | 0.14 | 0.27 | 0.12 | 1.10 |
| TG(16:1_38:5) | Triglycerides | 0.36 | 0.20 | 0.30 | 0.15 | 0.84 |
| TG(17:0_32:1) | Triglycerides | 0.27 | 0.46 | 0.31 | 0.22 | 1.15 |
| TG(17:0_34:1) | Triglycerides | 0.59 | 0.61 | 0.81 | 0.51 | 1.37 |
| TG(17:0_34:2) | Triglycerides | 0.59 | 0.37 | 0.73 | 0.39 | 1.23 |
| TG(17:0_34:3) | Triglycerides | 0.16 | 0.11 | 0.19 | 0.12 | 1.20 |
| TG(17:0_36:3) | Triglycerides | 0.87 | 0.39 | 1.03 | 0.52 | 1.19 |
| TG(17:0_36:4) | Triglycerides | 0.35 | 0.15 | 0.42 | 0.21 | 1.20 |
| TG(17:1_32:1) | Triglycerides | 0.38 | 0.86 | 0.54 | 0.68 | 1.41 |
| TG(17:1_34:1) | Triglycerides | 0.81 | 0.83 | 0.94 | 0.60 | 1.16 |
| TG(17:1_34:2) | Triglycerides | 0.53 | 0.37 | 0.75 | 0.61 | 1.42 |
| TG(17:1_34:3) | Triglycerides | 0.89 | 0.63 | 0.65 | 0.63 | 0.73 |
| TG(17:1_36:3) | Triglycerides | 0.50 | 0.23 | 0.53 | 0.26 | 1.06 |
| TG(17:1_36:4) | Triglycerides | 0.23 | 0.12 | 0.25 | 0.12 | 1.09 |
| TG(17:1_36:5) | Triglycerides | 0.08 | 0.04 | 0.11 | 0.05 | 1.38 |
| TG(17:1_38:5) | Triglycerides | 0.07 | 0.04 | 0.11 | 0.07 | 1.50 |
| TG(17:1_38:6) | Triglycerides | 0.06 | 0.04 | 0.09 | 0.06 | 1.44 |
| TG(17:1_38:7) | Triglycerides | 0.02 | 0.01 | 0.02 | 0.01 | 1.28 |
| TG(17:2_34:2) | Triglycerides | 0.13 | 0.09 | 0.14 | 0.08 | 1.06 |
| TG(17:2_34:3) | Triglycerides | 0.13 | 0.12 | 0.21 | 0.10 | 1.59 |
| TG(17:2_36:2) | Triglycerides | 0.18 | 0.11 | 0.20 | 0.20 | 1.16 |
| TG(17:2_36:3) | Triglycerides | 0.17 | 0.09 | 0.19 | 0.08 | 1.14 |
| TG(17:2_36:4) | Triglycerides | 0.37 | 0.21 | 0.47 | 0.26 | 1.26 |
| TG(17:2_38:5) | Triglycerides | 0.12 | 0.05 | 0.15 | 0.07 | 1.30 |
| TG(17:2_38:6) | Triglycerides | 0.17 | 0.08 | 0.20 | 0.09 | 1.18 |
| TG(17:2_38:7) | Triglycerides | 0.07 | 0.04 | 0.10 | 0.05 | 1.39 |
| TG(18:0_30:0) | Triglycerides | 0.96 | 2.57 | 0.24 | 0.27 | 0.25 |
| TG(18:0_30:1) | Triglycerides | 0.35 | 0.83 | 0.41 | 1.13 | 1.19 |
| TG(18:0_32:0) | Triglycerides | 1.12 | 2.71 | 0.80 | 0.83 | 0.72 |
| TG(18:0_32:1) | Triglycerides | 1.00 | 2.05 | 1.21 | 1.30 | 1.21 |
| TG(18:0_32:2) | Triglycerides | 0.36 | 0.54 | 0.54 | 0.52 | 1.51 |
| TG(18:0_34:2) | Triglycerides | 2.64 | 3.25 | 3.49 | 2.42 | 1.32 |
| TG(18:0_34:3) | Triglycerides | 0.68 | 0.62 | 0.89 | 0.57 | 1.32 |
| TG(18:0_36:1) | Triglycerides | 0.80 | 0.99 | 1.21 | 0.79 | 1.51 |
| TG(18:0_36:2) | Triglycerides | 3.67 | 2.75 | 5.22 | 3.71 | 1.42 |
| TG(18:0_36:3) | Triglycerides | 4.64 | 2.52 | 6.11 | 3.64 | 1.32 |
| TG(18:0_36:4) | Triglycerides | 2.11 | 1.19 | 2.63 | 1.62 | 1.25 |
| TG(18:0_36:5) | Triglycerides | 0.48 | 0.22 | 0.52 | 0.27 | 1.09 |
| TG(18:0_38:6) | Triglycerides | 0.41 | 0.17 | 0.64 | 0.37 | 1.57 |
| TG(18:0_38:7) | Triglycerides | 0.17 | 0.06 | 0.18 | 0.08 | 1.06 |
| TG(18:1_26:0) | Triglycerides | 0.41 | 1.01 | 0.85 | 1.71 | 2.06 |
| TG(18:1_28:1) | Triglycerides | 0.46 | 0.72 | 1.45 | 1.95 | 3.19 |
| TG(18:1_30:0) | Triglycerides | 4.32 | 6.06 | 6.14 | 7.24 | 1.42 |
| TG(18:1_30:1) | Triglycerides | 2.77 | 3.15 | 3.94 | 4.92 | 1.42 |
| TG(18:1_30:2) | Triglycerides | 0.69 | 0.71 | 1.08 | 1.15 | 1.57 |
| TG(18:1_31:0) | Triglycerides | 0.57 | 0.81 | 0.75 | 0.49 | 1.31 |
| TG(18:1_32:0) | Triglycerides | 16.72 | 23.86 | 24.52 | 17.94 | 1.47 |
| TG(18:1_32:1) | Triglycerides | 21.60 | 19.39 | 28.52 | 21.15 | 1.32 |
| TG(18:1_32:2) | Triglycerides | 6.07 | 3.63 | 7.48 | 5.02 | 1.23 |
| TG(18:1_32:3) | Triglycerides | 0.81 | 0.49 | 1.02 | 0.66 | 1.26 |
| TG(18:1_33:0) | Triglycerides | 0.60 | 0.71 | 0.91 | 0.60 | 1.52 |
| TG(18:1_33:1) | Triglycerides | 2.00 | 1.45 | 2.52 | 1.57 | 1.26 |
| TG(18:1_33:2) | Triglycerides | 0.85 | 0.48 | 0.97 | 0.49 | 1.14 |
| TG(18:1_33:3) | Triglycerides | 0.22 | 0.11 | 0.23 | 0.12 | 1.08 |
| TG(18:1_34:1) | Triglycerides | 104.59 | 74.48 | 141.74 | 83.66 | 1.36 |
| TG(18:1_34:2) | Triglycerides | 69.02 | 36.87 | 87.05 | 45.50 | 1.26 |
| TG(18:1_34:3) | Triglycerides | 13.23 | 6.28 | 14.46 | 7.05 | 1.09 |
| TG(18:1_34:4) | Triglycerides | 1.52 | 0.77 | 1.50 | 0.80 | 0.98 |
| TG(18:1_35:2) | Triglycerides | 1.59 | 0.74 | 2.04 | 0.95 | 1.28 |
| TG(18:1_35:3) | Triglycerides | 0.60 | 0.26 | 0.62 | 0.29 | 1.04 |
| TG(18:1_36:0) | Triglycerides | 1.08 | 0.94 | 1.61 | 1.02 | 1.49 |
| TG(18:1_36:1) | Triglycerides | 9.42 | 6.25 | 12.94 | 8.46 | 1.37 |
| TG(18:1_36:2) | Triglycerides | 34.59 | 18.86 | 43.54 | 24.89 | 1.26 |
| TG(18:1_36:3) | Triglycerides | 31.14 | 15.28 | 36.55 | 22.01 | 1.17 |
| TG(18:1_36:4) | Triglycerides | 12.22 | 6.65 | 13.93 | 9.57 | 1.14 |
| TG(18:1_36:5) | Triglycerides | 3.40 | 1.80 | 3.50 | 2.27 | 1.03 |
| TG(18:1_36:6) | Triglycerides | 0.65 | 0.44 | 0.80 | 0.84 | 1.23 |
| TG(18:1_38:5) | Triglycerides | 2.31 | 0.89 | 2.26 | 0.89 | 0.97 |
| TG(18:1_38:6) | Triglycerides | 2.40 | 1.24 | 1.60 | 0.93 | 0.67 |
| TG(18:1_38:7) | Triglycerides | 0.49 | 0.23 | 0.52 | 0.32 | 1.07 |
| TG(18:2_28:0) | Triglycerides | 0.54 | 0.94 | 0.89 | 1.35 | 1.64 |
| TG(18:2_30:0) | Triglycerides | 1.99 | 2.28 | 2.76 | 2.95 | 1.39 |
| TG(18:2_30:1) | Triglycerides | 1.74 | 1.18 | 1.69 | 1.79 | 0.97 |
| TG(18:2_31:0) | Triglycerides | 0.25 | 0.23 | 0.44 | 0.25 | 1.73 |
| TG(18:2_32:0) | Triglycerides | 8.93 | 9.34 | 12.46 | 8.58 | 1.40 |
| TG(18:2_32:1) | Triglycerides | 10.47 | 6.68 | 13.32 | 8.45 | 1.27 |
| TG(18:2_32:2) | Triglycerides | 2.77 | 1.51 | 3.24 | 2.20 | 1.17 |
| TG(18:2_33:0) | Triglycerides | 0.34 | 0.29 | 0.51 | 0.31 | 1.50 |
| TG(18:2_33:1) | Triglycerides | 1.04 | 0.57 | 1.41 | 0.70 | 1.35 |
| TG(18:2_33:2) | Triglycerides | 0.41 | 0.21 | 0.50 | 0.27 | 1.22 |
| TG(18:2_34:0) | Triglycerides | 6.59 | 4.76 | 8.71 | 4.88 | 1.32 |
| TG(18:2_34:1) | Triglycerides | 56.55 | 30.38 | 73.33 | 39.18 | 1.30 |
| TG(18:2_34:2) | Triglycerides | 33.63 | 16.78 | 40.23 | 22.34 | 1.20 |
| TG(18:2_34:3) | Triglycerides | 5.91 | 2.99 | 6.53 | 3.85 | 1.10 |
| TG(18:2_34:4) | Triglycerides | 0.70 | 0.38 | 0.73 | 0.44 | 1.05 |
| TG(18:2_35:1) | Triglycerides | 0.90 | 0.46 | 1.07 | 0.56 | 1.18 |
| TG(18:2_35:2) | Triglycerides | 0.83 | 0.40 | 0.97 | 0.51 | 1.17 |
| TG(18:2_35:3) | Triglycerides | 0.31 | 0.16 | 0.29 | 0.15 | 0.91 |
| TG(18:2_36:0) | Triglycerides | 0.60 | 0.39 | 0.79 | 0.45 | 1.32 |
| TG(18:2_36:1) | Triglycerides | 5.57 | 2.93 | 7.07 | 4.17 | 1.27 |
| TG(18:2_36:2) | Triglycerides | 17.40 | 8.52 | 20.64 | 12.51 | 1.19 |
| TG(18:2_36:3) | Triglycerides | 13.21 | 7.93 | 15.37 | 11.67 | 1.16 |
| TG(18:2_36:4) | Triglycerides | 5.76 | 4.47 | 6.33 | 5.70 | 1.10 |
| TG(18:2_36:5) | Triglycerides | 1.56 | 1.18 | 1.55 | 1.33 | 1.00 |
| TG(18:2_38:4) | Triglycerides | 0.59 | 0.26 | 0.68 | 0.26 | 1.16 |
| TG(18:2_38:5) | Triglycerides | 1.09 | 0.49 | 1.06 | 0.47 | 0.97 |
| TG(18:2_38:6) | Triglycerides | 1.30 | 0.79 | 1.01 | 0.60 | 0.78 |
| TG(18:3_30:0) | Triglycerides | 0.32 | 0.39 | 0.78 | 1.01 | 2.45 |
| TG(18:3_32:0) | Triglycerides | 1.19 | 1.34 | 2.28 | 2.56 | 1.92 |
| TG(18:3_32:1) | Triglycerides | 1.48 | 1.11 | 1.57 | 1.03 | 1.07 |
| TG(18:3_33:2) | Triglycerides | 0.08 | 0.05 | 0.12 | 0.08 | 1.46 |
| TG(18:3_34:0) | Triglycerides | 0.84 | 0.70 | 1.09 | 0.67 | 1.30 |
| TG(18:3_34:1) | Triglycerides | 7.16 | 4.50 | 8.45 | 5.13 | 1.18 |
| TG(18:3_34:2) | Triglycerides | 4.32 | 2.43 | 4.84 | 2.91 | 1.12 |
| TG(18:3_34:3) | Triglycerides | 0.85 | 0.49 | 0.73 | 0.43 | 0.86 |
| TG(18:3_35:2) | Triglycerides | 0.19 | 0.09 | 0.25 | 0.20 | 1.29 |
| TG(18:3_36:1) | Triglycerides | 0.82 | 0.49 | 1.02 | 0.62 | 1.24 |
| TG(18:3_36:2) | Triglycerides | 3.55 | 1.91 | 3.84 | 2.51 | 1.08 |
| TG(18:3_36:3) | Triglycerides | 3.10 | 1.88 | 3.27 | 2.40 | 1.06 |
| TG(18:3_36:4) | Triglycerides | 1.22 | 0.92 | 1.27 | 0.97 | 1.04 |
| TG(18:3_38:5) | Triglycerides | 0.21 | 0.11 | 0.19 | 0.18 | 0.94 |
| TG(18:3_38:6) | Triglycerides | 0.20 | 0.11 | 0.16 | 0.21 | 0.79 |
| TG(20:0_32:3) | Triglycerides | 0.25 | 0.14 | 0.33 | 0.15 | 1.34 |
| TG(20:0_32:4) | Triglycerides | 0.20 | 0.10 | 0.29 | 0.14 | 1.48 |
| TG(20:0_34:1) | Triglycerides | 0.13 | 0.11 | 0.16 | 0.07 | 1.21 |
| TG(20:1_24:3) | Triglycerides | N.D. | N.D. | N.D. | N.D. | N.D. |
| TG(20:1_26:1) | Triglycerides | 0.54 | 0.13 | N.D. | N.D. | N.D. |
| TG(20:1_30:1) | Triglycerides | 0.09 | 0.07 | 0.09 | 0.04 | 1.01 |
| TG(20:1_32:0) | Triglycerides | 0.06 | 0.08 | 0.06 | 0.03 | 0.96 |
| TG(20:1_32:1) | Triglycerides | 0.18 | 0.20 | 0.23 | 0.12 | 1.26 |
| TG(20:1_32:2) | Triglycerides | 0.13 | 0.07 | 0.14 | 0.05 | 1.06 |
| TG(20:1_32:3) | Triglycerides | 0.38 | 0.20 | 0.26 | 0.18 | 0.68 |
| TG(20:1_34:0) | Triglycerides | 0.10 | 0.07 | 0.16 | 0.14 | 1.49 |
| TG(20:1_34:1) | Triglycerides | 0.63 | 0.47 | 0.72 | 0.42 | 1.13 |
| TG(20:1_34:2) | Triglycerides | 0.51 | 0.34 | 0.56 | 0.30 | 1.09 |
| TG(20:1_34:3) | Triglycerides | 0.15 | 0.09 | 0.18 | 0.17 | 1.24 |
| TG(20:2_32:0) | Triglycerides | 0.16 | 0.26 | 0.29 | 0.32 | 1.83 |
| TG(20:2_32:1) | Triglycerides | 0.20 | 0.16 | 0.27 | 0.14 | 1.37 |
| TG(20:2_34:1) | Triglycerides | 0.56 | 0.35 | 0.74 | 0.37 | 1.32 |
| TG(20:2_34:2) | Triglycerides | 0.42 | 0.22 | 0.54 | 0.24 | 1.28 |
| TG(20:2_34:3) | Triglycerides | 0.16 | 0.07 | 0.22 | 0.15 | 1.40 |
| TG(20:2_34:4) | Triglycerides | 0.01 | 0.00 | 0.01 | 0.01 | 0.99 |
| TG(20:2_36:5) | Triglycerides | 0.07 | 0.02 | 0.08 | 0.03 | 1.09 |
| TG(20:3_32:0) | Triglycerides | 0.24 | 0.32 | 0.40 | 0.24 | 1.72 |
| TG(20:3_32:1) | Triglycerides | 0.26 | 0.22 | 0.35 | 0.19 | 1.36 |
| TG(20:3_32:2) | Triglycerides | 0.08 | 0.05 | 0.11 | 0.06 | 1.37 |
| TG(20:3_34:0) | Triglycerides | 0.21 | 0.14 | 0.42 | 0.37 | 1.95 |
| TG(20:3_34:1) | Triglycerides | 1.04 | 0.66 | 1.31 | 0.70 | 1.25 |
| TG(20:3_34:2) | Triglycerides | 0.70 | 0.39 | 0.87 | 0.37 | 1.24 |
| TG(20:3_34:3) | Triglycerides | 0.19 | 0.10 | 0.11 | 0.08 | 0.60 |
| TG(20:3_36:3) | Triglycerides | 0.33 | 0.15 | 0.37 | 0.14 | 1.13 |
| TG(20:3_36:4) | Triglycerides | 0.25 | 0.09 | 0.29 | 0.16 | 1.15 |
| TG(20:3_36:5) | Triglycerides | 0.15 | 0.04 | 0.15 | 0.10 | 1.00 |
| TG(20:4_30:0) | Triglycerides | 0.20 | 0.29 | 0.47 | 0.60 | 2.36 |
| TG(20:4_32:0) | Triglycerides | 0.61 | 0.83 | 0.95 | 0.57 | 1.56 |
| TG(20:4_32:1) | Triglycerides | 0.62 | 0.60 | 0.74 | 0.46 | 1.20 |
| TG(20:4_32:2) | Triglycerides | 0.18 | 0.11 | 0.31 | 0.22 | 1.67 |
| TG(20:4_33:2) | Triglycerides | 0.05 | 0.03 | 0.03 | 0.02 | 0.62 |
| TG(20:4_34:0) | Triglycerides | 0.48 | 0.37 | 0.85 | 0.74 | 1.77 |
| TG(20:4_34:1) | Triglycerides | 3.02 | 2.01 | 3.79 | 1.92 | 1.26 |
| TG(20:4_34:2) | Triglycerides | 2.00 | 1.15 | 2.46 | 1.12 | 1.23 |
| TG(20:4_34:3) | Triglycerides | 0.38 | 0.19 | 0.59 | 0.48 | 1.56 |
| TG(20:4_35:3) | Triglycerides | 0.03 | 0.02 | 0.02 | 0.01 | 0.49 |
| TG(20:4_36:2) | Triglycerides | 1.78 | 0.66 | 2.07 | 0.69 | 1.16 |
| TG(20:4_36:3) | Triglycerides | 1.10 | 0.46 | 1.22 | 0.44 | 1.11 |
| TG(20:4_36:4) | Triglycerides | 0.45 | 0.20 | 0.48 | 0.21 | 1.08 |
| TG(20:4_36:5) | Triglycerides | 0.15 | 0.08 | 0.12 | 0.06 | 0.77 |
| TG(20:5_34:0) | Triglycerides | 0.40 | 0.39 | 0.07 | 0.04 | 0.16 |
| TG(20:5_34:1) | Triglycerides | 2.33 | 1.94 | 1.85 | 1.31 | 0.80 |
| TG(20:5_34:2) | Triglycerides | 1.86 | 1.49 | 1.06 | 0.77 | 0.57 |
| TG(20:5_36:2) | Triglycerides | 1.59 | 1.16 | 0.71 | 0.46 | 0.45 |
| TG(20:5_36:3) | Triglycerides | 1.17 | 0.85 | 1.33 | 1.20 | 1.14 |
| TG(22:0_32:4) | Triglycerides | 0.06 | 0.13 | 0.06 | 0.04 | 1.00 |
| TG(22:1_32:5) | Triglycerides | 0.03 | 0.02 | 0.03 | 0.02 | 1.12 |
| TG(22:2_32:4) | Triglycerides | 0.06 | 0.04 | 0.09 | 0.06 | 1.42 |
| TG(22:3_30:2) | Triglycerides | 0.10 | 0.02 | 0.10 | 0.07 | 1.03 |
| TG(22:4_32:0) | Triglycerides | 0.07 | 0.08 | 0.14 | 0.08 | 2.06 |
| TG(22:4_32:2) | Triglycerides | 0.05 | 0.03 | 0.04 | 0.03 | 0.80 |
| TG(22:4_34:2) | Triglycerides | 0.20 | 0.11 | 0.40 | 0.26 | 2.01 |
| TG(22:5_32:0) | Triglycerides | 0.28 | 0.35 | 0.25 | 0.17 | 0.90 |
| TG(22:5_32:1) | Triglycerides | 0.45 | 0.33 | 0.34 | 0.22 | 0.76 |
| TG(22:5_34:1) | Triglycerides | 1.80 | 0.96 | 1.92 | 0.93 | 1.07 |
| TG(22:5_34:2) | Triglycerides | 1.37 | 0.69 | 1.01 | 0.50 | 0.74 |
| TG(22:5_34:3) | Triglycerides | 0.34 | 0.16 | 0.37 | 0.32 | 1.09 |
| TG(22:6_32:0) | Triglycerides | 1.81 | 1.89 | 2.31 | 2.31 | 1.27 |
| TG(22:6_32:1) | Triglycerides | 2.15 | 1.90 | 0.75 | 0.64 | 0.35 |
| TG(22:6_34:1) | Triglycerides | 8.93 | 6.12 | 7.26 | 5.50 | 0.81 |
| TG(22:6_34:2) | Triglycerides | 6.30 | 4.39 | 3.27 | 2.33 | 0.52 |
| TG(22:6_34:3) | Triglycerides | 1.57 | 1.27 | 1.59 | 1.75 | 1.01 |
| Thr | Amino acids | 126.25 | 27.30 | 120.31 | 37.45 | 0.95 |
| TLCA | Bile acids | 0.00 | 0.00 | 0.00 | 0.01 | 1.80 |
| TMAO | Amine oxides | 10.65 | 16.19 | 6.30 | 6.58 | 0.59 |
| TMCA | Bile acids | 0.00 | 0.00 | 0.01 | 0.01 | 2.19 |
| Trigonelline | Alkaloids | 1.00 | 1.44 | 0.82 | 0.98 | 0.82 |
| Trp | Amino acids | 51.80 | 8.77 | 47.98 | 11.16 | 0.93 |
| TrpBetaine | Amino acid related | 0.01 | 0.02 | 0.02 | 0.03 | 2.05 |
| Tyr | Amino acids | 62.95 | 13.08 | 60.59 | 16.05 | 0.96 |
| Val | Amino acids | 231.31 | 42.48 | 235.56 | 57.97 | 1.02 |
| Xanthine | Nucleobases and related | 0.45 | 0.25 | 0.45 | 0.41 | 1.00 |

N.D. represents not determined.

**Table S2**

Significantly increased metabolites in the plasma of endometrial cancer patients

| Metabolite | Class | Cancer (µM, mean ± SD) | Cohort (µM, mean ± SD) | *p*-value | FDR | Fold change (Cancer/Cohort) |
| --- | --- | --- | --- | --- | --- | --- |
| Cystine | Amino acid related | 125.43 ± 32.84 | 24.53 ± 12.42 | 2.99E-108 | 1.25E-105 | 5.11 |
| Hypoxanthine | Nucleobases and related | 9.46 ± 21.68 | 2.62 ± 1.47 | 0.0001096 | 0.00041469 | 3.65 |
| C8 | Acylcarnitines | 0.52 ± 0.53 | 0.20 ± 0.06 | 1.81E-11 | 2.38E-10 | 2.46 |
| beta-Ala | Biogenic amines | 0.11 ± 0.08 | 0.05 ± 0.04 | 6.76E-18 | 1.89E-16 | 2.41 |
| TG(18:3_30:0) | Triglycerides | 0.78 ± 1.01 | 0.32 ± 0.39 | 6.53E-07 | 3.71E-06 | 2.15 |
| GUDCA | Bile acids | 0.51 ± 1.15 | 0.26 ± 0.39 | 0.012471 | 0.022872 | 1.95 |
| C5-OH (C3-DC-M) | Acylcarnitines | 0.05 ± 0.02 | 0.03 ± 0.01 | 2.94E-31 | 2.47E-29 | 1.66 |
| TG(18:2_28:0) | Triglycerides | 0.89 ± 1.35 | 0.54 ± 0.94 | 0.015896 | 0.027819 | 1.60 |
| TG(16:0_34:0) | Triglycerides | 5.14 ± 3.99 | 3.24 ± 6.44 | 0.0028282 | 0.0065266 | 1.58 |
| TG(18:0_36:1) | Triglycerides | 1.21 ± 0.79 | 0.80 ± 0.99 | 3.46E-05 | 0.00016328 | 1.56 |
| TG(16:0_35:1) | Triglycerides | 0.86 ± 0.61 | 0.58 ± 0.83 | 0.00039764 | 0.0012652 | 1.53 |
| GCDCA | Bile acids | 0.67 ± 0.71 | 0.44 ± 0.61 | 0.0035167 | 0.0076928 | 1.51 |
| TG(18:1_33:0) | Triglycerides | 0.91 ± 0.60 | 0.60 ± 0.71 | 0.00012471 | 0.00046354 | 1.51 |
| TG(18:1_36:0) | Triglycerides | 1.61 ± 1.02 | 1.08 ± 0.94 | 1.02E-05 | 5.20E-05 | 1.47 |
| TG(18:2_33:0) | Triglycerides | 0.51 ± 0.31 | 0.34 ± 0.29 | 6.99E-06 | 3.62E-05 | 1.47 |
| TG(18:1_32:0) | Triglycerides | 24.52 ± 17.94 | 16.72 ± 23.86 | 0.0017585 | 0.0045027 | 1.47 |
| TG(16:0_34:1) | Triglycerides | 41.07 ± 30.99 | 28.05 ± 42.43 | 0.0029999 | 0.0068105 | 1.46 |
| TG(20:4_34:3) | Triglycerides | 0.59 ± 0.48 | 0.38 ± 0.19 | 2.50E-06 | 1.36E-05 | 1.45 |
| TG(18:0_32:2) | Triglycerides | 0.54 ± 0.52 | 0.36 ± 0.54 | 0.0041933 | 0.0087621 | 1.45 |
| TG(18:1_30:0) | Triglycerides | 6.14 ± 7.24 | 4.32 ± 6.06 | 0.014081 | 0.025383 | 1.45 |
| C16:1 | Acylcarnitines | 0.10 ± 0.06 | 0.07 ± 0.02 | 1.21E-09 | 1.08E-08 | 1.45 |
| TG(16:0_35:2) | Triglycerides | 1.41 ± 0.95 | 0.98 ± 0.86 | 7.42E-05 | 0.00029414 | 1.43 |
| TG(18:0_38:6) | Triglycerides | 0.64 ± 0.37 | 0.41 ± 0.17 | 2.07E-08 | 1.61E-07 | 1.43 |
| TG(14:0_36:1) | Triglycerides | 1.27 ± 1.19 | 0.85 ± 0.90 | 0.0027277 | 0.0063294 | 1.43 |
| TG(18:0_36:2) | Triglycerides | 5.22 ± 3.71 | 3.67 ± 2.75 | 5.28E-05 | 0.00023082 | 1.42 |
| TG(18:1_30:1) | Triglycerides | 3.94 ± 4.92 | 2.77 ± 3.15 | 0.015367 | 0.027185 | 1.42 |
| TG(16:0_34:2) | Triglycerides | 35.61 ± 25.97 | 25.27 ± 26.97 | 0.00090697 | 0.0025061 | 1.41 |
| TG(14:0_34:1) | Triglycerides | 4.82 ± 5.58 | 3.47 ± 5.07 | 0.024628 | 0.040885 | 1.41 |
| TG(16:0_32:3) | Triglycerides | 0.76 ± 0.77 | 0.54 ± 0.60 | 0.0071918 | 0.014522 | 1.40 |
| TG(18:2_32:0) | Triglycerides | 12.46 ± 8.58 | 8.93 ± 9.34 | 0.00083387 | 0.0023664 | 1.40 |
| TG(18:2_30:0) | Triglycerides | 2.76 ± 2.95 | 1.99 ± 2.28 | 0.011184 | 0.020785 | 1.39 |
| TG(16:0_32:2) | Triglycerides | 3.93 ± 4.19 | 2.89 ± 3.84 | 0.019271 | 0.033036 | 1.38 |
| TG(17:0_34:1) | Triglycerides | 0.81 ± 0.51 | 0.59 ± 0.61 | 0.00075971 | 0.0022005 | 1.38 |
| TG(20:0_32:4) | Triglycerides | 0.29 ± 0.14 | 0.20 ± 0.10 | 1.33E-07 | 8.61E-07 | 1.38 |
| TG(20:4_32:0) | Triglycerides | 0.95 ± 0.57 | 0.61 ± 0.83 | 0.0045364 | 0.009432 | 1.38 |
| TG(14:0_34:2) | Triglycerides | 3.26 ± 3.24 | 2.37 ± 2.57 | 0.008858 | 0.016988 | 1.38 |
| TG(18:1_36:1) | Triglycerides | 12.94 ± 8.46 | 9.42 ± 6.25 | 5.54E-05 | 0.00023606 | 1.37 |
| TG(18:1_30:2) | Triglycerides | 1.08 ± 1.15 | 0.69 ± 0.71 | 0.01074 | 0.020048 | 1.37 |
| TG(16:0_36:2) | Triglycerides | 84.55 ± 49.51 | 62.32 ± 44.03 | 5.60E-05 | 0.00023606 | 1.36 |
| TG(18:1_34:1) | Triglycerides | 141.74 ± 83.66 | 104.59 ± 74.48 | 6.79E-05 | 0.00027159 | 1.36 |
| TG(16:0_37:3) | Triglycerides | 0.31 ± 0.16 | 0.23 ± 0.16 | 3.35E-05 | 0.00015992 | 1.35 |
| TG(16:1_34:1) | Triglycerides | 13.94 ± 10.85 | 10.51 ± 11.48 | 0.0088157 | 0.016984 | 1.33 |
| TG(18:0_34:2) | Triglycerides | 3.49 ± 2.42 | 2.64 ± 3.25 | 0.011788 | 0.021811 | 1.32 |
| TG(18:2_34:0) | Triglycerides | 8.71 ± 4.88 | 6.59 ± 4.76 | 0.00018927 | 0.00066801 | 1.32 |
| TG(18:1_32:1) | Triglycerides | 28.52 ± 21.15 | 21.60 ± 19.39 | 0.0035879 | 0.0077524 | 1.32 |
| TG(18:0_36:3) | Triglycerides | 6.11 ± 3.64 | 4.64 ± 2.52 | 5.97E-05 | 0.00024575 | 1.32 |
| TG(18:2_36:0) | Triglycerides | 0.79 ± 0.45 | 0.60 ± 0.39 | 0.0001305 | 0.00048079 | 1.32 |
| TG(18:0_34:3) | Triglycerides | 0.89 ± 0.57 | 0.68 ± 0.62 | 0.0023552 | 0.0056204 | 1.31 |
| ProBetaine | Amino acid related | 4.10 ± 4.20 | 3.12 ± 3.10 | 0.022539 | 0.037866 | 1.31 |
| TG(16:0_34:3) | Triglycerides | 10.89 ± 7.29 | 8.32 ± 6.70 | 0.0017506 | 0.0045027 | 1.31 |
| TG(18:2_33:1) | Triglycerides | 1.41 ± 0.70 | 1.04 ± 0.57 | 3.79E-05 | 0.00017559 | 1.30 |
| TG(18:2_34:1) | Triglycerides | 73.33 ± 39.18 | 56.55 ± 30.38 | 4.65E-05 | 0.00021008 | 1.30 |
| TG(14:0_36:2) | Triglycerides | 6.15 ± 4.85 | 4.74 ± 3.31 | 0.0036321 | 0.0077524 | 1.30 |
| TG(14:0_34:3) | Triglycerides | 0.88 ± 0.73 | 0.66 ± 0.56 | 0.010074 | 0.019061 | 1.30 |
| TG(16:0_36:3) | Triglycerides | 79.11 ± 42.43 | 61.48 ± 33.62 | 8.79E-05 | 0.00034195 | 1.29 |
| TG(20:2_34:1) | Triglycerides | 0.74 ± 0.37 | 0.56 ± 0.35 | 0.00017068 | 0.0006127 | 1.29 |
| TG(16:1_32:2) | Triglycerides | 0.76 ± 0.60 | 0.68 ± 0.79 | 0.026087 | 0.042799 | 1.28 |
| TG(16:0_38:3) | Triglycerides | 1.35 ± 0.74 | 1.05 ± 0.63 | 0.00021358 | 0.00073385 | 1.28 |
| TG(18:3_34:0) | Triglycerides | 1.09 ± 0.67 | 0.84 ± 0.70 | 0.0036796 | 0.0078051 | 1.28 |
| TG(16:0_35:3) | Triglycerides | 0.62 ± 0.36 | 0.49 ± 0.33 | 0.00079211 | 0.002267 | 1.28 |
| TG(18:2_32:1) | Triglycerides | 13.32 ± 8.45 | 10.47 ± 6.68 | 0.001348 | 0.0035834 | 1.27 |
| TG(18:2_36:1) | Triglycerides | 7.07 ± 4.17 | 5.57 ± 2.93 | 0.00037566 | 0.0012231 | 1.27 |
| TG(18:1_33:1) | Triglycerides | 2.52 ± 1.57 | 2.00 ± 1.45 | 0.0028815 | 0.0065773 | 1.26 |
| TG(17:2_36:4) | Triglycerides | 0.47 ± 0.26 | 0.37 ± 0.21 | 0.00046935 | 0.0014495 | 1.26 |
| TG(18:1_34:2) | Triglycerides | 87.05 ± 45.50 | 69.02 ± 36.87 | 0.00020589 | 0.00072062 | 1.26 |
| DG(16:0_18:2) | Diglycerides | 1.11 ± 0.65 | 0.86 ± 0.52 | 0.0011893 | 0.0031816 | 1.26 |
| TG(18:1_36:2) | Triglycerides | 43.54 ± 24.89 | 34.59 ± 18.86 | 0.00053699 | 0.0016226 | 1.26 |
| TG(20:4_34:1) | Triglycerides | 3.79 ± 1.92 | 3.02 ± 2.01 | 0.00084577 | 0.0023841 | 1.26 |
| TG(18:1_35:2) | Triglycerides | 2.04 ± 0.95 | 1.59 ± 0.74 | 6.44E-05 | 0.0002602 | 1.25 |
| TG(18:0_36:4) | Triglycerides | 2.63 ± 1.62 | 2.11 ± 1.19 | 0.0017689 | 0.0045027 | 1.25 |
| TG(20:2_32:1) | Triglycerides | 0.27 ± 0.14 | 0.20 ± 0.16 | 0.0053177 | 0.011002 | 1.24 |
| TG(20:2_34:2) | Triglycerides | 0.54 ± 0.24 | 0.42 ± 0.22 | 0.00021491 | 0.00073385 | 1.24 |
| TG(17:2_38:5) | Triglycerides | 0.15 ± 0.07 | 0.12 ± 0.05 | 5.07E-05 | 0.0002267 | 1.23 |
| TG(20:4_34:2) | Triglycerides | 2.46 ± 1.12 | 2.00 ± 1.15 | 0.00053465 | 0.0016226 | 1.23 |
| TG(18:1_32:2) | Triglycerides | 7.48 ± 5.02 | 6.07 ± 3.63 | 0.0057337 | 0.011772 | 1.23 |
| TG(20:3_34:1) | Triglycerides | 1.31 ± 0.70 | 1.04 ± 0.66 | 0.0028745 | 0.0065773 | 1.23 |
| TG(17:0_34:2) | Triglycerides | 0.73 ± 0.39 | 0.59 ± 0.37 | 0.0025216 | 0.0059166 | 1.23 |
| TG(16:0_36:4) | Triglycerides | 26.46 ± 15.15 | 21.61 ± 11.52 | 0.0020003 | 0.0049832 | 1.22 |
| TG(14:0_36:3) | Triglycerides | 5.61 ± 4.09 | 4.59 ± 2.65 | 0.010112 | 0.019061 | 1.22 |
| TG(18:2_33:2) | Triglycerides | 0.50 ± 0.27 | 0.41 ± 0.21 | 0.0017297 | 0.0044844 | 1.22 |
| TG(20:0_32:3) | Triglycerides | 0.33 ± 0.15 | 0.25 ± 0.14 | 0.0016697 | 0.0043829 | 1.22 |
| DG(16:0_18:1) | Diglycerides | 1.82 ± 1.15 | 1.47 ± 1.11 | 0.015405 | 0.027185 | 1.22 |
| TG(18:3_36:1) | Triglycerides | 1.02 ± 0.62 | 0.82 ± 0.49 | 0.0073748 | 0.01475 | 1.21 |
| TG(16:0_38:4) | Triglycerides | 1.40 ± 0.80 | 1.16 ± 0.76 | 0.0086482 | 0.016794 | 1.21 |
| TG(17:0_34:3) | Triglycerides | 0.19 ± 0.12 | 0.16 ± 0.11 | 0.016593 | 0.028917 | 1.20 |
| TG(18:1_32:3) | Triglycerides | 1.02 ± 0.66 | 0.81 ± 0.49 | 0.015822 | 0.027804 | 1.20 |
| TG(18:2_34:2) | Triglycerides | 40.23 ± 22.34 | 33.63 ± 16.78 | 0.0041847 | 0.0087621 | 1.20 |
| TG(16:1_34:2) | Triglycerides | 8.96 ± 5.58 | 7.52 ± 5.67 | 0.02868 | 0.046689 | 1.19 |
| TG(18:2_36:2) | Triglycerides | 20.64 ± 12.51 | 17.40 ± 8.52 | 0.0091561 | 0.01748 | 1.19 |
| TG(17:0_36:3) | Triglycerides | 1.03 ± 0.52 | 0.87 ± 0.39 | 0.0026553 | 0.0061958 | 1.19 |
| DG(18:1_18:1) | Diglycerides | 1.88 ± 0.88 | 1.56 ± 0.79 | 0.0035975 | 0.0077524 | 1.18 |
| TG(18:2_35:1) | Triglycerides | 1.07 ± 0.56 | 0.90 ± 0.46 | 0.0057458 | 0.011772 | 1.18 |
| TG(18:3_34:1) | Triglycerides | 8.45 ± 5.13 | 7.16 ± 4.50 | 0.021606 | 0.036444 | 1.18 |
| DG(18:1_18:2) | Diglycerides | 4.21 ± 1.89 | 3.53 ± 1.58 | 0.0020529 | 0.0050423 | 1.18 |
| TG(17:0_36:4) | Triglycerides | 0.42 ± 0.21 | 0.35 ± 0.15 | 0.0032216 | 0.0071972 | 1.18 |
| TG(18:1_36:3) | Triglycerides | 36.55 ± 22.01 | 31.14 ± 15.28 | 0.013996 | 0.025337 | 1.17 |
| TG(18:2_35:2) | Triglycerides | 0.97 ± 0.51 | 0.83 ± 0.40 | 0.0085221 | 0.016648 | 1.17 |
| TG(20:4_36:2) | Triglycerides | 2.07 ± 0.69 | 1.78 ± 0.66 | 0.00029971 | 0.00098344 | 1.16 |
| C14 | Acylcarnitines | 0.05 ± 0.02 | 0.04 ± 0.02 | 0.0024319 | 0.0057381 | 1.15 |
| Cortisol | Hormones and related | 0.23 ± 0.09 | 0.20 ± 0.07 | 0.0020463 | 0.0050423 | 1.15 |
| TG(18:2_38:4) | Triglycerides | 0.68 ± 0.26 | 0.59 ± 0.26 | 0.0034924 | 0.0076796 | 1.15 |
| Glu | Amino acids | 56.81 ± 30.96 | 49.34 ± 15.84 | 0.0086769 | 0.016794 | 1.15 |
| PC aa C32:1 | Phosphatidylcholines | 12.77 ± 6.78 | 11.13 ± 5.70 | 0.02503 | 0.041225 | 1.15 |
| TG(17:2_38:6) | Triglycerides | 0.20 ± 0.09 | 0.17 ± 0.08 | 0.023937 | 0.040054 | 1.13 |
| Ile | Amino acids | 69.64 ± 20.68 | 62.28 ± 14.78 | 0.00045593 | 0.0014184 | 1.12 |
| SM C20:2 | Sphingomyelins | 0.24 ± 0.07 | 0.22 ± 0.07 | 0.007671 | 0.015269 | 1.11 |
| C9 | Acylcarnitines | 0.04 ± 0.01 | 0.04 ± 0.01 | 0.008447 | 0.016578 | 1.10 |
| Arg | Amino acids | 78.92 ± 24.69 | 71.64 ± 22.48 | 0.0083523 | 0.016536 | 1.10 |
| Pro | Amino acids | 152.23 ± 47.10 | 138.65 ± 39.33 | 0.0073513 | 0.01475 | 1.10 |
| Kynurenine | Amino acid related | 1.68 ± 0.53 | 1.56 ± 0.44 | 0.024091 | 0.040152 | 1.08 |
| HexCer(d18:1/16:0) | Hexosylceramides | 1.16 ± 0.30 | 1.08 ± 0.22 | 0.017736 | 0.030782 | 1.07 |

**Table S3**

Significantly decreased metabolites in the plasma of endometrial cancer patients.

| Metabolite | Class | Cancer (µM, mean ± SD) | Cohort (µM, mean ± SD) | *p*-value | FDR | Fold change (Cancer/Cohort) |
| --- | --- | --- | --- | --- | --- | --- |
| TG(16:0_40:8) | Triglycerides | 0.59 ± 0.42 | 3.76 ± 5.76 | 2.56E-10 | 2.69E-09 | 0.16 |
| TG(22:6_32:1) | Triglycerides | 0.75 ± 0.64 | 2.15 ± 1.90 | 2.29E-15 | 5.35E-14 | 0.35 |
| TG(16:0_38:7) | Triglycerides | 0.43 ± 0.28 | 1.15 ± 1.53 | 2.57E-07 | 1.52E-06 | 0.39 |
| TG(20:5_36:2) | Triglycerides | 0.71 ± 0.46 | 1.59 ± 1.16 | 1.52E-15 | 3.76E-14 | 0.45 |
| SM C26:1 | Sphingomyelins | 0.13 ± 0.07 | 0.29 ± 0.10 | 3.12E-40 | 4.37E-38 | 0.46 |
| TG(22:6_34:2) | Triglycerides | 3.27 ± 2.33 | 6.30 ± 4.39 | 2.17E-12 | 3.51E-11 | 0.52 |
| Serotonin | Biogenic amines | 0.14 ± 0.11 | 0.27 ± 0.20 | 1.59E-11 | 2.23E-10 | 0.52 |
| SM C26:0 | Sphingomyelins | 0.05 ± 0.01 | 0.09 ± 0.03 | 1.75E-41 | 3.67E-39 | 0.56 |
| TG(20:5_34:2) | Triglycerides | 1.06 ± 0.77 | 1.86 ± 1.49 | 1.80E-08 | 1.43E-07 | 0.56 |
| CE(22:6) | Cholesteryl esters | 26.37 ± 12.38 | 46.59 ± 16.24 | 8.03E-26 | 4.22E-24 | 0.59 |
| TMAO | Amine oxides | 6.30 ± 6.58 | 10.65 ± 16.19 | 0.0031101 | 0.0070229 | 0.59 |
| DHA | Fatty acids | 60.56 ± 44.61 | 101.15 ± 61.58 | 4.66E-10 | 4.55E-09 | 0.60 |
| EPA | Fatty acids | 5.78 ± 6.47 | 9.15 ± 7.33 | 3.80E-05 | 0.00017559 | 0.63 |
| PC ae C38:1 | Phosphatidylcholines | 0.67 ± 0.20 | 1.05 ± 0.29 | 1.36E-31 | 1.43E-29 | 0.64 |
| TG(16:0_40:7) | Triglycerides | 1.54 ± 1.02 | 2.39 ± 1.79 | 1.10E-06 | 6.05E-06 | 0.64 |
| HArg | Amino acid related | 3.41 ± 1.55 | 5.22 ± 1.93 | 1.08E-16 | 2.83E-15 | 0.65 |
| TG(18:1_38:6) | Triglycerides | 1.60 ± 0.93 | 2.40 ± 1.24 | 1.26E-09 | 1.10E-08 | 0.66 |
| lysoPC a C17:0 | Lysophosphatidylcholines | 1.32 ± 0.43 | 1.94 ± 0.54 | 3.40E-23 | 1.43E-21 | 0.68 |
| lysoPC a C18:0 | Lysophosphatidylcholines | 26.71 ± 8.54 | 38.96 ± 9.27 | 1.49E-26 | 9.90E-25 | 0.69 |
| CE(20:5) | Cholesteryl esters | 59.09 ± 58.00 | 83.85 ± 59.63 | 6.40E-05 | 0.0002602 | 0.70 |
| BABA | Amino acid related | 0.16 ± 0.08 | 0.23 ± 0.04 | 8.61E-19 | 2.78E-17 | 0.70 |
| Hex3Cer(d18:1/18:0) | Trihexosylceramides | 0.13 ± 0.05 | 0.19 ± 0.05 | 1.61E-20 | 5.62E-19 | 0.70 |
| C18:2 | Acylcarnitines | 0.07 ± 0.03 | 0.10 ± 0.04 | 1.71E-11 | 2.31E-10 | 0.71 |
| PC aa C24:0 | Phosphatidylcholines | 0.11 ± 0.05 | 0.15 ± 0.08 | 2.27E-08 | 1.71E-07 | 0.71 |
| lysoPC a C16:0 | Lysophosphatidylcholines | 86.01 ± 23.20 | 119.15 ± 25.03 | 1.65E-26 | 9.90E-25 | 0.72 |
| PC aa C42:6 | Phosphatidylcholines | 0.44 ± 0.21 | 0.60 ± 0.28 | 3.85E-08 | 2.74E-07 | 0.73 |
| TG(22:5_34:2) | Triglycerides | 1.01 ± 0.50 | 1.37 ± 0.69 | 2.28E-07 | 1.37E-06 | 0.73 |
| TG(22:5_32:1) | Triglycerides | 0.34 ± 0.22 | 0.45 ± 0.33 | 0.0003818 | 0.0012335 | 0.74 |
| lysoPC a C26:0 | Lysophosphatidylcholines | 0.28 ± 0.07 | 0.38 ± 0.15 | 3.35E-12 | 5.22E-11 | 0.74 |
| SM (OH) C22:2 | Sphingomyelins | 4.48 ± 1.04 | 6.04 ± 1.32 | 1.68E-24 | 7.82E-23 | 0.74 |
| lysoPC a C14:0 | Lysophosphatidylcholines | 1.27 ± 0.50 | 1.70 ± 0.57 | 2.70E-11 | 3.34E-10 | 0.74 |
| PC aa C40:2 | Phosphatidylcholines | 0.28 ± 0.12 | 0.37 ± 0.18 | 5.84E-07 | 3.36E-06 | 0.75 |
| PC aa C36:6 | Phosphatidylcholines | 1.09 ± 0.45 | 1.44 ± 0.59 | 3.66E-08 | 2.65E-07 | 0.76 |
| lysoPC a C16:1 | Lysophosphatidylcholines | 2.30 ± 0.82 | 3.01 ± 0.76 | 2.40E-13 | 4.59E-12 | 0.76 |
| PC aa C40:3 | Phosphatidylcholines | 0.46 ± 0.17 | 0.60 ± 0.20 | 5.26E-10 | 5.02E-09 | 0.77 |
| lysoPC a C24:0 | Lysophosphatidylcholines | 0.22 ± 0.05 | 0.28 ± 0.09 | 3.36E-14 | 7.05E-13 | 0.77 |
| Cer(d16:1/24:0) | Ceramides | 0.12 ± 0.05 | 0.15 ± 0.06 | 9.24E-08 | 6.16E-07 | 0.77 |
| lysoPC a C26:1 | Lysophosphatidylcholines | 0.41 ± 0.10 | 0.54 ± 0.17 | 2.24E-13 | 4.48E-12 | 0.77 |
| TG(18:2_38:6) | Triglycerides | 1.01 ± 0.60 | 1.30 ± 0.79 | 0.00044996 | 0.0014103 | 0.78 |
| C18:1 | Acylcarnitines | 0.16 ± 0.05 | 0.20 ± 0.05 | 6.74E-13 | 1.18E-11 | 0.78 |
| PC aa C42:5 | Phosphatidylcholines | 0.28 ± 0.10 | 0.36 ± 0.13 | 9.11E-09 | 7.36E-08 | 0.78 |
| Hex2Cer(d18:1/20:0) | Dihexosylceramides | 0.09 ± 0.02 | 0.12 ± 0.04 | 2.30E-09 | 1.90E-08 | 0.78 |
| lysoPC a C18:1 | Lysophosphatidylcholines | 17.63 ± 6.21 | 22.53 ± 5.11 | 1.09E-12 | 1.84E-11 | 0.78 |
| HexCer(d18:2/22:0) | Hexosylceramides | 0.62 ± 0.23 | 0.78 ± 0.21 | 6.53E-11 | 7.41E-10 | 0.78 |
| TG(16:0_36:6) | Triglycerides | 0.41 ± 0.29 | 0.52 ± 0.45 | 0.013347 | 0.024267 | 0.79 |
| PC ae C38:0 | Phosphatidylcholines | 1.98 ± 0.61 | 2.51 ± 0.75 | 8.63E-11 | 9.53E-10 | 0.79 |
| PC ae C42:1 | Phosphatidylcholines | 0.25 ± 0.05 | 0.31 ± 0.07 | 2.34E-18 | 7.02E-17 | 0.79 |
| TG(20:5_34:1) | Triglycerides | 1.85 ± 1.31 | 2.33 ± 1.94 | 0.014644 | 0.02606 | 0.80 |
| PC aa C42:4 | Phosphatidylcholines | 0.12 ± 0.02 | 0.15 ± 0.03 | 4.53E-23 | 1.73E-21 | 0.80 |
| PC aa C42:2 | Phosphatidylcholines | 0.21 ± 0.06 | 0.27 ± 0.07 | 8.69E-12 | 1.30E-10 | 0.80 |
| PC ae C42:2 | Phosphatidylcholines | 0.38 ± 0.09 | 0.47 ± 0.10 | 7.16E-15 | 1.58E-13 | 0.81 |
| Hex3Cer(d18:1/22:0) | Trihexosylceramides | 0.17 ± 0.05 | 0.21 ± 0.07 | 6.40E-08 | 4.41E-07 | 0.81 |
| lysoPC a C28:0 | Lysophosphatidylcholines | 0.51 ± 0.12 | 0.63 ± 0.17 | 6.13E-11 | 7.15E-10 | 0.81 |
| PC aa C36:0 | Phosphatidylcholines | 1.65 ± 0.63 | 2.03 ± 0.67 | 9.23E-07 | 5.17E-06 | 0.81 |
| TG(22:6_34:1) | Triglycerides | 7.26 ± 5.50 | 8.93 ± 6.12 | 0.014537 | 0.025981 | 0.81 |
| Orn | Amino acid related | 76.06 ± 28.88 | 93.52 ± 23.39 | 2.39E-08 | 1.76E-07 | 0.81 |
| PC aa C36:5 | Phosphatidylcholines | 33.90 ± 18.23 | 41.55 ± 23.97 | 0.0023358 | 0.0056073 | 0.82 |
| PC aa C40:6 | Phosphatidylcholines | 33.06 ± 9.56 | 40.48 ± 13.65 | 1.57E-07 | 9.83E-07 | 0.82 |
| Cit | Amino acid related | 29.05 ± 7.82 | 35.56 ± 8.95 | 1.50E-10 | 1.61E-09 | 0.82 |
| PC ae C40:1 | Phosphatidylcholines | 1.12 ± 0.31 | 1.37 ± 0.31 | 2.32E-11 | 2.96E-10 | 0.82 |
| TG(18:3_34:3) | Triglycerides | 0.73 ± 0.43 | 0.85 ± 0.49 | 0.0036113 | 0.0077524 | 0.82 |
| Taurine | Amino acid related | 61.33 ± 20.95 | 74.61 ± 20.95 | 1.09E-07 | 7.15E-07 | 0.82 |
| Hex3Cer(d18:1/24:1) | Trihexosylceramides | 0.58 ± 0.17 | 0.70 ± 0.21 | 5.19E-08 | 3.63E-07 | 0.82 |
| SM (OH) C22:1 | Sphingomyelins | 5.78 ± 1.46 | 7.02 ± 1.60 | 3.00E-11 | 3.59E-10 | 0.82 |
| Cer(d18:1/26:1) | Ceramides | 0.03 ± 0.01 | 0.03 ± 0.01 | 5.36E-05 | 0.00023188 | 0.83 |
| Hex2Cer(d18:1/22:0) | Dihexosylceramides | 0.19 ± 0.05 | 0.23 ± 0.06 | 1.08E-09 | 9.90E-09 | 0.83 |
| FA(20:2) | Fatty acids | 0.75 ± 0.44 | 0.91 ± 0.65 | 0.010121 | 0.019061 | 0.83 |
| C14:1 | Acylcarnitines | 0.07 ± 0.03 | 0.08 ± 0.04 | 0.0002702 | 0.00090068 | 0.84 |
| PC aa C38:0 | Phosphatidylcholines | 2.92 ± 0.76 | 3.50 ± 0.81 | 1.46E-09 | 1.25E-08 | 0.84 |
| Cer(d18:2/24:0) | Ceramides | 0.33 ± 0.11 | 0.39 ± 0.12 | 2.73E-06 | 1.47E-05 | 0.84 |
| TG(16:1_38:5) | Triglycerides | 0.30 ± 0.15 | 0.36 ± 0.20 | 0.0064991 | 0.013187 | 0.84 |
| Cer(d18:1/14:0) | Ceramides | 0.06 ± 0.03 | 0.06 ± 0.03 | 0.00063215 | 0.0018567 | 0.84 |
| Cer(d16:1/18:0) | Ceramides | 0.10 ± 0.05 | 0.11 ± 0.05 | 0.0010698 | 0.0028989 | 0.84 |
| FA(18:1) | Fatty acids | 127.68 ± 58.77 | 152.41 ± 62.63 | 0.00065257 | 0.0019033 | 0.84 |
| PC ae C30:2 | Phosphatidylcholines | 0.14 ± 0.03 | 0.16 ± 0.04 | 4.22E-10 | 4.22E-09 | 0.84 |
| SM (OH) C24:1 | Sphingomyelins | 0.56 ± 0.12 | 0.67 ± 0.16 | 9.18E-10 | 8.57E-09 | 0.84 |
| PC aa C38:6 | Phosphatidylcholines | 102.65 ± 27.89 | 121.92 ± 33.99 | 2.18E-07 | 1.33E-06 | 0.84 |
| PC ae C38:6 | Phosphatidylcholines | 6.34 ± 1.74 | 7.47 ± 1.78 | 7.77E-08 | 5.26E-07 | 0.85 |
| HCys | Amino acid related | 2.75 ± 1.21 | 3.23 ± 1.12 | 0.00050893 | 0.0015602 | 0.85 |
| Cer(d16:1/20:0) | Ceramides | 0.12 ± 0.06 | 0.14 ± 0.05 | 0.00085937 | 0.0024062 | 0.85 |
| PC aa C38:5 | Phosphatidylcholines | 44.35 ± 12.94 | 52.12 ± 17.42 | 2.06E-05 | 0.00010167 | 0.85 |
| PC ae C38:2 | Phosphatidylcholines | 1.63 ± 0.39 | 1.91 ± 0.46 | 2.26E-08 | 1.71E-07 | 0.85 |
| C2 | Acylcarnitines | 7.13 ± 3.03 | 8.37 ± 3.10 | 0.0005878 | 0.0017509 | 0.85 |
| PC ae C44:3 | Phosphatidylcholines | 0.12 ± 0.02 | 0.14 ± 0.03 | 1.17E-11 | 1.70E-10 | 0.85 |
| PC ae C32:2 | Phosphatidylcholines | 0.69 ± 0.19 | 0.81 ± 0.19 | 1.80E-07 | 1.11E-06 | 0.86 |
| Cer(d18:2/23:0) | Ceramides | 0.10 ± 0.03 | 0.11 ± 0.04 | 0.0001568 | 0.00056774 | 0.86 |
| PC ae C40:6 | Phosphatidylcholines | 3.91 ± 1.00 | 4.55 ± 1.04 | 1.36E-07 | 8.64E-07 | 0.86 |
| PC ae C42:3 | Phosphatidylcholines | 0.64 ± 0.14 | 0.74 ± 0.13 | 3.74E-10 | 3.83E-09 | 0.86 |
| PC aa C40:5 | Phosphatidylcholines | 6.35 ± 2.04 | 7.37 ± 2.47 | 0.00014282 | 0.0005216 | 0.86 |
| PC ae C40:5 | Phosphatidylcholines | 2.28 ± 0.50 | 2.65 ± 0.51 | 1.73E-09 | 1.45E-08 | 0.86 |
| Cer(d16:1/23:0) | Ceramides | 0.08 ± 0.03 | 0.09 ± 0.04 | 0.0020051 | 0.0049832 | 0.86 |
| His | Amino acids | 73.33 ± 15.73 | 85.00 ± 10.11 | 2.94E-13 | 5.37E-12 | 0.86 |
| lysoPC a C28:1 | Lysophosphatidylcholines | 0.55 ± 0.15 | 0.63 ± 0.17 | 6.08E-06 | 3.20E-05 | 0.86 |
| CE(18:3) | Cholesteryl esters | 18.02 ± 8.12 | 20.38 ± 7.04 | 0.0023364 | 0.0056073 | 0.87 |
| PC ae C34:3 | Phosphatidylcholines | 5.22 ± 1.67 | 6.00 ± 1.62 | 5.82E-05 | 0.00024222 | 0.87 |
| lysoPC a C20:4 | Lysophosphatidylcholines | 5.19 ± 1.72 | 5.95 ± 1.46 | 5.27E-05 | 0.00023082 | 0.87 |
| AconAcid | Carboxylic acids | 10.27 ± 2.62 | 11.77 ± 3.12 | 1.26E-05 | 6.38E-05 | 0.87 |
| Choline | Vitamins and cofactors | 10.55 ± 3.27 | 12.06 ± 2.74 | 2.13E-05 | 0.00010383 | 0.87 |
| Cer(d16:1/22:0) | Ceramides | 0.19 ± 0.07 | 0.22 ± 0.08 | 0.0022021 | 0.0053771 | 0.88 |
| CE(18:1) | Cholesteryl esters | 68.82 ± 21.42 | 78.03 ± 19.31 | 9.49E-05 | 0.00036549 | 0.88 |
| Gly | Amino acids | 226.01 ± 75.13 | 255.79 ± 87.51 | 0.0019373 | 0.0048722 | 0.88 |
| Cer(d18:2/22:0) | Ceramides | 0.22 ± 0.06 | 0.25 ± 0.08 | 0.00057564 | 0.0017269 | 0.89 |
| PC aa C28:1 | Phosphatidylcholines | 3.48 ± 0.87 | 3.93 ± 0.97 | 3.88E-05 | 0.00017708 | 0.89 |
| PC ae C40:2 | Phosphatidylcholines | 1.44 ± 0.33 | 1.62 ± 0.39 | 2.40E-05 | 0.00011562 | 0.89 |
| PC ae C30:1 | Phosphatidylcholines | 2.08 ± 0.71 | 2.34 ± 0.71 | 0.0017903 | 0.0045296 | 0.89 |
| Asp | Amino acids | 7.13 ± 3.01 | 7.98 ± 1.85 | 0.0034533 | 0.0076335 | 0.89 |
| PC ae C36:3 | Phosphatidylcholines | 4.47 ± 1.14 | 4.99 ± 1.09 | 8.74E-05 | 0.00034195 | 0.90 |
| HexCer(d18:2/24:0) | Hexosylceramides | 0.85 ± 0.27 | 0.94 ± 0.27 | 0.0022915 | 0.0055631 | 0.90 |
| GABA | Biogenic amines | 0.15 ± 0.04 | 0.17 ± 0.03 | 1.65E-05 | 8.23E-05 | 0.90 |
| PC ae C36:0 | Phosphatidylcholines | 0.90 ± 0.25 | 1.00 ± 0.34 | 0.0038231 | 0.0080688 | 0.90 |
| lysoPC a C20:3 | Lysophosphatidylcholines | 1.62 ± 0.71 | 1.80 ± 0.59 | 0.020253 | 0.034314 | 0.90 |
| PC aa C34:4 | Phosphatidylcholines | 1.10 ± 0.45 | 1.22 ± 0.39 | 0.014363 | 0.02578 | 0.90 |
| Ser | Amino acids | 110.76 ± 26.03 | 122.73 ± 24.33 | 5.62E-05 | 0.00023606 | 0.90 |
| PC ae C36:1 | Phosphatidylcholines | 5.69 ± 1.39 | 6.29 ± 1.35 | 0.00021257 | 0.00073385 | 0.90 |
| PC aa C32:3 | Phosphatidylcholines | 0.46 ± 0.11 | 0.50 ± 0.10 | 0.00011976 | 0.00044911 | 0.91 |
| PC aa C42:1 | Phosphatidylcholines | 0.29 ± 0.08 | 0.32 ± 0.06 | 0.00060807 | 0.0017985 | 0.91 |
| PC aa C40:4 | Phosphatidylcholines | 1.58 ± 0.48 | 1.74 ± 0.46 | 0.0036362 | 0.0077524 | 0.91 |
| Cer(d18:1/24:0) | Ceramides | 1.59 ± 0.47 | 1.75 ± 0.45 | 0.0031542 | 0.0070844 | 0.91 |
| PC ae C40:3 | Phosphatidylcholines | 0.75 ± 0.14 | 0.83 ± 0.14 | 6.09E-06 | 3.20E-05 | 0.91 |
| SM C24:0 | Sphingomyelins | 11.58 ± 2.73 | 12.73 ± 2.61 | 0.00026385 | 0.00088654 | 0.91 |
| PC aa C38:3 | Phosphatidylcholines | 31.09 ± 7.90 | 34.16 ± 8.39 | 0.0013706 | 0.0036204 | 0.91 |
| PC ae C36:5 | Phosphatidylcholines | 7.20 ± 2.01 | 7.91 ± 1.67 | 0.0010661 | 0.0028989 | 0.91 |
| PC ae C34:2 | Phosphatidylcholines | 7.47 ± 1.85 | 8.20 ± 1.85 | 0.00079345 | 0.002267 | 0.91 |
| lysoPC a C18:2 | Lysophosphatidylcholines | 27.56 ± 11.20 | 30.18 ± 9.38 | 0.029725 | 0.048203 | 0.91 |
| C0 | Acylcarnitines | 47.44 ± 10.22 | 51.89 ± 10.36 | 0.00024772 | 0.00083904 | 0.91 |
| SM C24:1 | Sphingomyelins | 30.25 ± 6.02 | 33.05 ± 6.66 | 0.00018151 | 0.00064605 | 0.92 |
| Cer(d18:2/24:1) | Ceramides | 0.22 ± 0.06 | 0.24 ± 0.06 | 0.001724 | 0.0044844 | 0.92 |
| PC ae C40:4 | Phosphatidylcholines | 1.30 ± 0.29 | 1.41 ± 0.26 | 0.0003966 | 0.0012652 | 0.92 |
| PC ae C38:3 | Phosphatidylcholines | 2.36 ± 0.52 | 2.57 ± 0.49 | 0.0004302 | 0.0013585 | 0.92 |
| Gln | Amino acids | 585.17 ± 87.00 | 636.98 ± 83.33 | 3.19E-07 | 1.86E-06 | 0.92 |
| Met | Amino acids | 22.01 ± 5.69 | 23.95 ± 4.19 | 0.0009143 | 0.0025098 | 0.92 |
| PC ae C30:0 | Phosphatidylcholines | 0.33 ± 0.09 | 0.35 ± 0.10 | 0.01185 | 0.021829 | 0.92 |
| Hex3Cer(d18:1/16:0) | Trihexosylceramides | 1.29 ± 0.29 | 1.40 ± 0.33 | 0.0023966 | 0.0056868 | 0.92 |
| CE(20:3) | Cholesteryl esters | 6.12 ± 1.78 | 6.39 ± 2.00 | 0.018672 | 0.032272 | 0.92 |
| SM (OH) C14:1 | Sphingomyelins | 3.31 ± 0.81 | 3.59 ± 0.91 | 0.005805 | 0.011835 | 0.92 |
| SM C16:0 | Sphingomyelins | 79.67 ± 14.24 | 86.19 ± 16.15 | 0.00028114 | 0.00092974 | 0.92 |
| PC aa C36:1 | Phosphatidylcholines | 37.82 ± 11.26 | 40.84 ± 11.00 | 0.020261 | 0.034314 | 0.93 |
| Trp | Amino acids | 47.98 ± 11.16 | 51.80 ± 8.77 | 0.0011403 | 0.00307 | 0.93 |
| PC ae C32:1 | Phosphatidylcholines | 2.11 ± 0.50 | 2.28 ± 0.48 | 0.0034093 | 0.0075763 | 0.93 |
| PC ae C34:0 | Phosphatidylcholines | 1.04 ± 0.30 | 1.12 ± 0.30 | 0.02001 | 0.034163 | 0.93 |
| C18 | Acylcarnitines | 0.04 ± 0.01 | 0.04 ± 0.01 | 0.024773 | 0.040962 | 0.93 |
| PC ae C38:5 | Phosphatidylcholines | 10.73 ± 2.17 | 11.54 ± 1.97 | 0.00088245 | 0.0024545 | 0.93 |
| HexCer(d18:1/24:1) | Hexosylceramides | 4.09 ± 1.02 | 4.40 ± 0.98 | 0.0083863 | 0.016536 | 0.93 |
| HexCer(d18:1/24:0) | Hexosylceramides | 1.51 ± 0.42 | 1.62 ± 0.45 | 0.026677 | 0.043596 | 0.93 |
| ADMA | Amino acid related | 0.46 ± 0.08 | 0.50 ± 0.07 | 0.00010761 | 0.00041087 | 0.93 |
| PC aa C36:2 | Phosphatidylcholines | 175.28 ± 44.09 | 187.76 ± 41.83 | 0.013041 | 0.023814 | 0.93 |
| SM (OH) C16:1 | Sphingomyelins | 1.92 ± 0.45 | 2.05 ± 0.51 | 0.018865 | 0.032473 | 0.94 |
| SM C16:1 | Sphingomyelins | 11.52 ± 2.30 | 12.22 ± 2.40 | 0.010631 | 0.019933 | 0.94 |
