# Supplementary figures and images for: Identification of predictive biomarkers for endometrial cancer diagnosis and treatment response monitoring using plasma metabolome profiling
Source: Cancer Metab. 2023 Oct 11;11:16. doi: 10.1186/s40170-023-00317-z (PMC10568780; doi:10.1186/s40170-023-00317-z)

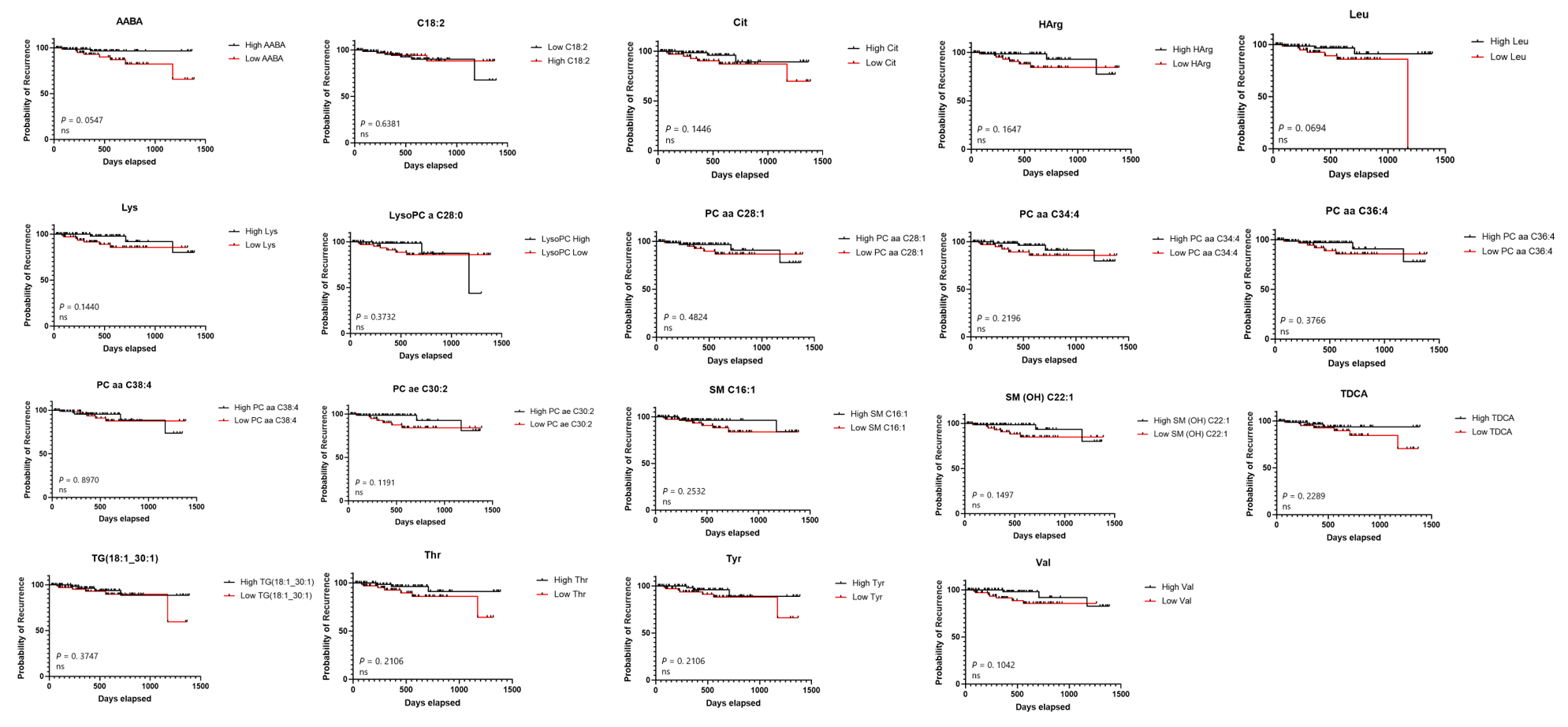

Supplement: Supplementary file 2 — Additional file 2: Fig. S1. Kaplan-Meier survival analysis of metabolites correlated with risk of recurrence in EMC patients. The Kaplan-Meier survival analysis divided the patients into two groups according to the median metabolite concentration and compared the days of recurrence using the log-rank test. The black line shows Group 1, the red line shows Group 2, the vertical axis shows probability of recurrence and the horizontal axis shows days of elapse. Shown for 19 metabolites that are not significant between the two groups. [file 40170_2023_317_MOESM2_ESM.tif]
